# Prediction of an MMP-1 inhibitor activity cliff using the SAR matrix approach and its experimental validation

**DOI:** 10.1038/s41598-020-71696-2

**Published:** 2020-09-07

**Authors:** Yasunobu Asawa, Atsushi Yoshimori, Jürgen Bajorath, Hiroyuki Nakamura

**Affiliations:** 1 Laboratory for Chemistry and Life Science, Institute of Innovative Research, Tokyo Institute of Technology, Nagatsuta-cho, Midori-ku, Yokohama 226-8503 Japan; 2 School of Life Science and Technology, Tokyo Institute of Technology, Nagatsuta-cho, Midori-ku, Yokohama 226-8503 Japan; 3 Institute for Theoretical Medicine, Inc., 26-1, Muraoka-Higashi 2-chome, Fujisawa, Kanagawa 251-0012 Japan; 4 Department of Life Science Informatics, B-IT, LIMES Program Unit Chemical Biology and Medicinal Chemistry, Rheinische Friedrich-Wilhelms-Universität, Endenicher Allee 19c, 53115 Bonn, Germany

**Keywords:** Oncology, Cheminformatics, Medicinal chemistry, Organic chemistry, Chemical synthesis, Drug discovery, Medicinal chemistry

## Abstract

A matrix metalloproteinase 1 (MMP-1) inhibitor activity cliff was predicted using the SAR Matrix method. Compound **4** was predicted as a highly potent activity cliff partner and found to possess 60 times higher inhibitory activity against MMP-1 than the structurally related compound **3**. Furthermore, pharmacophore fitting of synthesized compounds indicated that the correctly predicted activity cliff was caused by interactions between the trifluoromethyl group at para position in compound **4** and residue ARG214 of MMP-1.

## Introduction

Structure activity relationship (SAR) analysis plays an important role in lead optimization to predict increasingly potent analogues. Generally, in the presence of SAR continuity, compound modifications lead to gradual changes in potency^1^. In this case, QSAR methods can be applied for potency prediction of analogues. In contrast, in the presence of SAR discontinuity, small chemical modifications of active compounds might cause significant changes in potency and lead to the formation of activity cliffs^2,3^. In such cases, QSAR methods are not applicable and compound potency is difficult to predict^4,5^. However, existing SAR data might be thoroughly analyzed to predict novel active compounds.

The SAR Matrix (SARM) methodology was developed for systematic analysis of SAR data sets and the prediction of virtual analogues of known active compounds. SARM organizes analogue series and associated SAR information in matrices on the basis of structural relationships between series and reveals activity cliffs^6,7^. Following the SARM approach, the existing compounds are fragmented in a size-restricted manner by systematic cleavage of exocyclic single bonds. These procedures result in a two-dimensional of core structure and substituent fragments and unexplored combinations of such fragments (virtual analogues). The activity of virtual analogues can be predicted using SARM-based local Free-Wilson models. Previously, it was demonstrated that such predictions can identify novel active compounds^8,9^. Furthermore, SARM modeling and associated local potency predictions might conceivably also be applied to predict new activity cliffs formed by existing compounds and virtual analogs. Herein, we report the SARM-based prediction and experimental verification of a matrix metalloproteinase 1 (MMP-1) inhibitor activity cliff formed by a known inhibitor and a virtual analogue originating from SARM.

## Results and discussion

**Figure 1:**
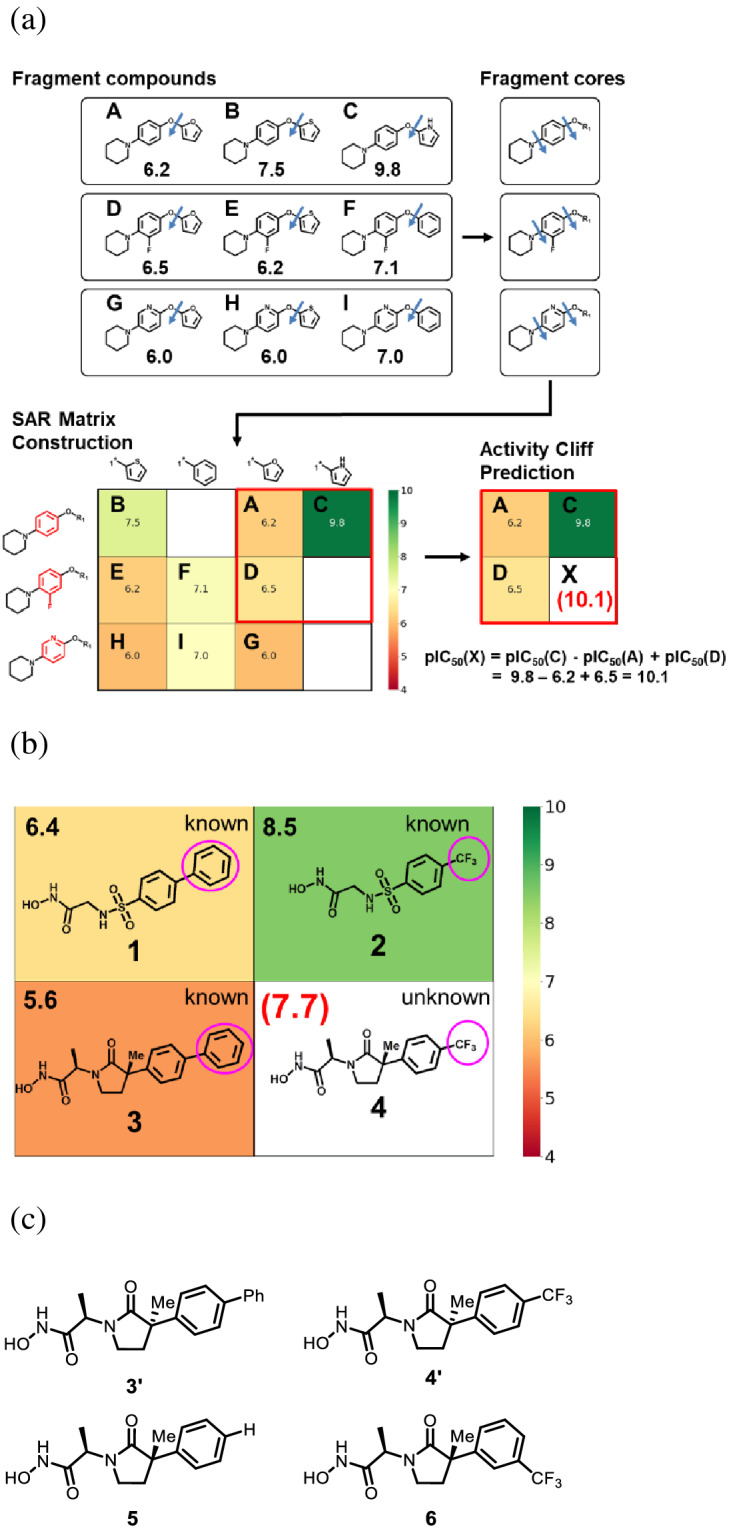
Activity cliff prediction using the SAR matrix (SARM) method. (**a**) Schematic summary of SARM modeling. (**b**) Prediction of an activity cliff formed by MMP-1 inhibitors. (**c**) Structure of designed control compounds.

MMPs, one of the important collagenase families for degrading native collagen, play a central role in all major stages of tumor progression^10,11^. Although various MMP inhibitors have been considered as potential anti-cancer agents, only one drug, doxycycline, has been approved by the US Food and Drug Administration (FDA) for a different therapeutic application, i.e., the prevention of periodontitis^12^. In addition, another compound has entered phase II trials for Kaposi’s sarcoma and brain tumors^10,13^. Currently, a variety of MMP-1 inhibitors and their SAR data are available in the ChEMBL database^14^, which provided the basis of our SARM application and activity cliff prediction. To construct SARMs for MMP-1 inhibitors, we first obtained 644 compounds with available K_i_ values from ChEMBL (data set ChEMBL332). The MMP-1 inhibitor set yielded 2,697 individual SARMs that were searched for regions of SAR discontinuity as described^7^, (i.e., regions existing analogues have large potency variations). In these regions, the potency of virtual analogues was predicted using local Free-Wilson models, as illustrated in Fig. 1a. Compound environments were inpected for predictions that would yield activity cliffs formed by weakly potent known inhibitors and virtual analogues predicted to be much more portent. Only a limited number of such putative activity cliff constellations were identified and we concentrated on a chemically attractive example where a phenyl ring in a weakly potent inhibitor was replaced by a trifluoromethyl group in a virtual analogue, shown in Fig. 1b.

Compounds **1**^15,16^, **2**^17^, and **3**^18^ were known MMP-1 inhibitors included in the ChEMBL data sets. By contrast, virtual compound **4** originated from SARMs, representing a novel combination of a core and substituent extracted from structurally distinct inhibitors, as shown in Fig. 1b. It is emphasized that this compound **4** could not have been predicted using conventional QSAR methods on the basis of compound 1, which contains a distinct core structure and substituent, or compound **2**, which contains a distinct core. Moreover, the activity cliff formed by compounds **3** and **4** could not possibly be predicted on the basis of compound **3** alone because the prediction fully depended on the local SARM environment of virtual analogue **4**, as also illustrated in Fig. 1b. The potency of compound **4** was predicted to be at least one order of magnitude higher than of compound **3**. This prediction was particularly attractive because the potency of compound **2** was improved ~ 10-fold by a corresponding replacement of the phenyl group in compound **1** with a trifluoromethyl group. Hence, the formation of an activity cliff by compound **3** and its virtual analogue compound **4** was predicted. We also emphasize that the prediction did not depend on prior SAR knowledge or subjective intervention. Instead, the SARM approach systematically generates all matrix neighborhoods containing existing and virtual compounds that are amenable to potency predictions and automatically prioritizes activity cliffs on the basis of potency differences between existing and virtual analogs, as illustrated in Fig. 1b. The formation of activity cliffs generally is a rare event in compound data sets^4,5^ and in the case of MMP1 inhibitors, large numbers of known active compounds and new virtual analogues had to be systematically evaluated to predict the formation of an activity cliff formed by compounds **3** and **4.**

On the basis of the prediction, we synthesized compound **4**. In addition, compounds **3′** and **4′**, which were the diastereomers of **3** and **4**, respectively, were also synthesized in order to investigate the effect of stereochemical differences on the activity. Compound **5**^18^, in which the phenyl group of **3** was replaced by hydrogen atom, and compound **6,** in which the trifluoromethyl group of **4** was substituted at meta position, were also synthesized as control compounds for comparison (Fig. 1c).

Synthesis of compounds **3**–**6** is summarized in Scheme 1.18 Esters **7**–**10** were chosen as starting materials to be treated with lithium diisopropyl amide (LDA) in THF to introduce methyl and allyl groups stepwise at α position of each ester, and the resulting allylic esters were converted to the corresponding aldehydes **11**–**14** by ozonolysis. Reductive amination of the aldehydes **11**–**14** with D-alanine methyl ester followed by lactamization was carried out in the presence of zinc dust in acetic acid under reflux conditions in one pot to give the corresponding γ-lactams **15**–**18** in 29–49% yields with a 1:1 diastereomer ratio. After the diastereomers were separated by chromatography, γ-lactams **15**–**18** were converted into *N*-hydroxyamides **3**–**6** using NH_2_OH and KOH in 43–98% yields.

We next examined the inhibitory activity of the synthesized compounds **3**–**6** against MMP-1 using a colorimetric assay. This assay was performed using the MMP-1 Inhibitor Screening Assay Kit (ab139443) according to the manufacturer’s protocols. The reaction was started by the addition of the diluted MMP-1 substrate. The continuous absorbance of the wells was measured at A_412nm_ using a microplate reader. The results are summarized in Table 1. The IC_50_ value of compound **4** (IC_50_ = 0.18 ± 0.03 µM) was 60-fold lower than that of compound **3** (IC_50_ = 11.5 ± 1.3 µM), hence confirming the formation of the predicted activity cliff. In contrast, both diastereomers **3′** and **4′** did not display significant inhibitory activity even at a 100 µM concentration. On the other hand, the inhibitory activity of compound **5**, which had no substituent at the phenyl ring, was moderate (IC_50_ = 1.54 ± 0.08 µM), indicating that the trifluoromethyl substituent is more favorable than the phenyl group of compound **3**, probably due to improved steric/hydrophobic compatibility. Compound **6**, which had trifluoromethyl group at meta position, exhibited similar potency (IC_50_ = 11.1 ± 0.5 µM) to compound **3**.

**Table 1:** MMP-1 inhibitory activity of synthesized compounds.

Compound	IC_50_ [µM]^a^
3	11.5 ± 1.3
4	0.18 ± 0.03
5	1.54 ± 0.08
6	11.1 ± 0.5
3’	> 100
4’	> 100

^a^The compound concentration required for 50% inhibition (IC_50_) was determined from semi-logarithmic dose–response plots, and the results represent the mean ± standard deviation of triplicated samples.

**Figure 2:**
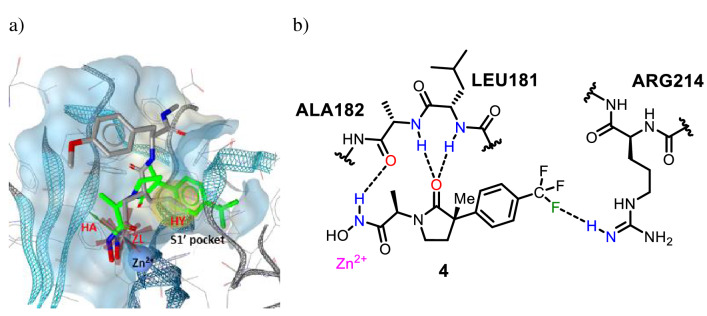
(**a**) Pharmacophore fitting of compound **4** to the SC44463 pharmacophore model; (**b**) Binding interaction of compound **4** with MMP-1.

**Scheme 1:**
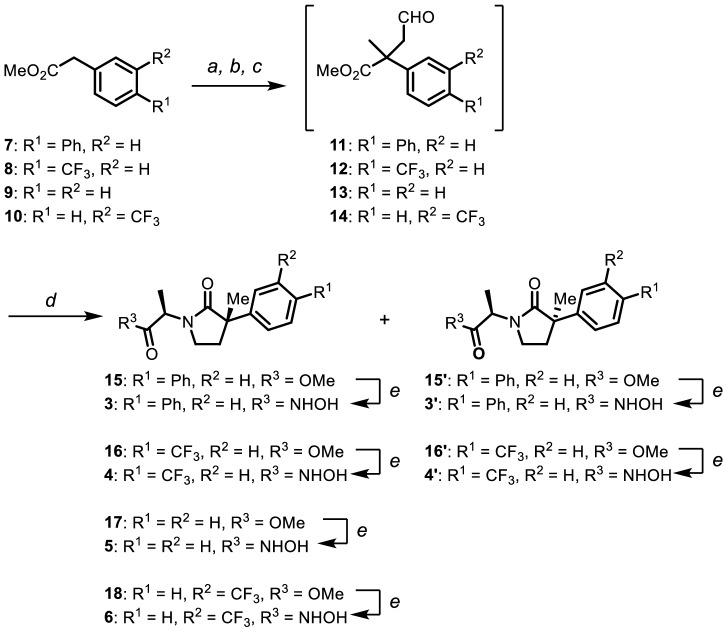
Synthesis of compounds **3**–**6. **Conditions: (**a**) LDA, MeI, THF, − 78 °C; (**b**) LDA, allyl bromide, THF, − 78 °C; (**c**) O_3_, CH_2_Cl_2_, PPh_3_ − 78 °C to r.t.; (**d**) _D_-alanine methyl ester, Zn, acetic acid, reflux (**15**: 29%; **16**: 47%; **17**: 49%; **18**: 29% yield for 4 steps; d.r. = 1:1); (**e**) NH_2_OH, MeOH (**3**: 72%; **3′**: 98%; **4**: 90%; **4′**: 72%; **5**: 49%; **6**: 43% yield).

To evaluate possible binding interactions between compound **4** and MMP-1, a pharmacophore model was constructed^19^ from the crystal structure of compound SC44463 in complex with MMP-1 (PDB entry 1FBL). SC44463 is a substrate-based inhibitor with a hydroxamic acid moiety, which chelates the active site zinc cation in MMP-1. Here, three pharmacophore features of SC44463 were used including a hydrogen bond acceptor (HA), hydrophobic moiety (Hy), and zinc binding location features (ZL) (Fig. 2a). The zinc-chelating and hydrophobic interactions with the S1′ pocket, which is sequence-variable within the MMP family, are related to potency and selectivity of MMP inhibitors^19,20^. Although compounds synthesized in Scheme 1 were racemic, the (*R*,*S*)-enantiomer of compound **4** was superimposed on SC44463 pharmacophore model and putative interactions were refined (Fig. 2b).

As a result, the trifluoromethyl phenyl group of compound **4** was placed into the hydrophobic S1′ pocket where the isobutyl group of SC44463 was located. Considering this model, the biphenyl group of compound **3** was thought to be too bulky to optimally match the S1′ pocket (see Figure S1 of the supporting information). Also, compound **4** might form a halogen bonding interaction involving the trifluoromethyl group and residue ARG214 of MMP-1 (with a calculated distance of ~ 2.5 Å; see Figure S2a) in the supporting information for details). On the other hand, both (*R*,*R*)-diastereomers **3′** and **4′**, exhibited significantly lower inhibitory activity toward MMP-1 (IC_50_ > 100 µM) than (*R*,*S*)-isomers **3** and **4**. In fact, the carbonyl group of the γ-lactam is expected to play an important role for the MMP-3 inhibitory activity through hydrogen bond formation with the backbone of residues LEU164 and ALA165^18^. Consistent with this notion, our model suggested hydrogen bond formation between the γ-lactam of compound **4** with LEU181 and ALA182 in MMP-1 (with calculated length of ~ 2.9 and ~ 2.8 Å, respectively; see Figure S2b of the supporting information for details), whereas this hydrogen bond formation could not be observed for isomer **4′** (Figure S1).

## Conclusion

We have investigated activity cliff formation based on SARM analysis targeting MMP-1 inhibitors. Compound **4** was selected as a virtual candidate for activity cliff formation on the basis of a thorough search using SARMs. Subsequently, compound **4** and derivatives were synthesized and examined for MMP-1 inhibitory activity. The predicted compound **4** was found to exhibit 60-fold higher potency than its analogue compound **3**, thereby confirming the predicted activity cliff. Retrospective pharmacophore analysis was consistent with the prediction and experimental observations, indicating a prominent interaction between the trifluoromethyl group at para position of compound **4** and ARG214 of MMP-1. Our case study and proof-of-concept investigation suggests that SARM-based analysis of compounds and associated SAR data extends the spectrum of compound design methods and enables the prediction of potent compounds and activity cliffs, representing scientifically stimulating and practically relevant applications. Further studies will aim to investigate the generalization potential and compound design perspectives suggested by this work. Specifically, our proof-of-concept study indicates that SARM-based virtual analogue populations of compound data sets can be systematically screened for predicted activity cliffs, without subjective intervention, which would not be possible using contemporary QSAR methods. Thus, prediction of activity cliffs can be attempted on a large scale for compounds with any biological activity.

## Methods

### General

Compounds **3** and **5** were known and synthesized according to the literature procedures^18^. Purity of the compounds tested for MMP-1 inhibitory assay was determined by HPLC analysis using Inertsil ODS-3 5 µm (4.6 × 75 mm; GL Science) with a linear gradient of 0.1% formic acid in water/0.1% formic acid in MeCN (100/0 to 0/100 for 10 min).

Synthesis of methyl (2*R*)-2-(3-methyl-2-oxo-3-(4-(trifluoromethyl)phenyl)pyrrolidin-1-yl)propanoate (16)

To a solution of methyl 2-(4-(trifluoromethyl)phenyl)acetate (**8**) (579 mg, 2.56 mmol) in THF (7.5 mL), was slowly added LDA 1.0 M solution in hexane/THF (1 : 2) (3.30 mL, 3.30 mmol) at − 78 °C. After the resulting mixture was stirred at 0 °C under argon atmosphere for 1 h, MeI (319 µL, 5.12 mmol) was added. Then, the resulting mixture was stirred at room temperature for 1 h. After that, the reaction mixture was concentrated under pressure. Brine was added to the mixture and the product was extracted with ethyl acetate, dried over sodium sulfate, and concentrated under vacuum. The crude product was used in the next step without further purification.

To a solution of the crude material in THF (7.5 mL), x LDA 1.0 M solution in hexane/THF (1 : 2) (3.30 mL, 3.30 mmol) at − 78 °C was slowly added. After the mixture was stirred at 0 °C under argon atmosphere for 1 h, allyl bromide (433 µL, 5.12 mmol) was added. The resulting mixture was stirred at room temperature for 1 h and the reaction mixture was concentrated under reduced pressure. The reaction was quenched with brine and the mixture was extracted with EtOAc, washed with hexane, dried over sodium sulfate, and concentrated under vacuum. The crude product was used in the next step without further purification.

Ozone was pumped into a –78 °C solution of the above crude material in CH_2_Cl_2_ (5 mL) until the starting material disappeared, as monitored by TLC analysis. The mixture was purged with argon. Triphenylphosphine (806 mg, 3.07 mmol) was added. After 1 h at room temperature, the mixture was concentrated under vacuum. Purification by short column chromatography on silica gel (20% EtOAc in Hexane) gave the crude product **12** which was used to the next step without further purification.

To a solution of the above product (**12**) and D-alanine methyl ester hydrochloride (275 mg, 1.97 mmol) in acetic acid (10.7 mL) was added portion-wise zinc powder (1.17 g, 17.9 mmol). The mixture was heated to reflux for 12 h, and then cooled to room temperature. Following addition of CH_2_Cl_2_, the mixture was filtered and the filter cake washed with methanol/ CH_2_Cl_2_. The filtrate was concentrated at 45 °C in vacuo to remove acetic acid. The residue was treated with ethyl acetate and filtered to remove insoluble materials. The filtrate was concentrated and purified by column chromatography on silica gel (40% EtOAc in hexane) to afford 179 mg of fast eluting isomer (**16′**), 135 mg of slow eluting isomer (**16**), and 80 mg of mixture containing a **mixture of both isomers (total** 394 mg, 1.20 mmol, 47% yield for 4 steps) as a colorless oil.

**16**: ^1^H NMR (400 MHz; CDCl_3_): δ 7.60 (d, *J* = 8.8 Hz, 2H), 7.56 ( *J* = 8.8 Hz, 2H), 4.97 (q, *J* = 7.5 Hz, 1H), 3.69 (s, 3H), 3.41 (q, *J* = 4.5 Hz, 2H), 2.48–2.41 (m, 1H), 2.23–2.16 (m, 1H), 1.58 (s, 3H), 1.48 (d, *J* = 7.2 Hz, 3H); ^13^C NMR (125 MHz; CDCl_3_): δ 177.3, 171.9, 147.9, 129.1 (q, *J* = 32.3 Hz), 126.7, 125.6 (q, *J* = 180.1 Hz), 125.5 (q, *J* = 3.6 Hz), 52.5, 49.8, 48.9, 40.6, 35.3, 24.5, 15.0; ^19^F NMR (470 MHz, CDCl_3_): δ 62.5; HRMS (ESI, positive) for C_16_H_18_F_3_NO_3_ (m/z): calculated 352.1131 (M + Na)^+^, found 352.1128.

**16′**: ^1^H NMR (400 MHz; CDCl_3_): δ 7.59 (d, *J* = 8.5 Hz, 2H), 7.56 (d, *J* = 8.5 Hz, 2H), 4.93 (q, *J* = 7.5 Hz, 1H), 3.75 (s, 3H), 3.53–3.49 (m, 1H), 3.36–3.30 (m, 1H), 2.46–2.39 (m, 1H), 2.30–2.23 (m, 1H), 1.58 (s, 3H), 1.45 (d, *J* = 7.5 Hz, 3H); ^13^C NMR (125 MHz; CDCl_3_): δ 177.3, 171.9, 147.8, 129.0 (q, *J* = 14.9 Hz), 126.7, 125.5 (q, *J* = 3.6 Hz), 124.2 (q, *J* = 270.2 Hz), 52.4, 49.8, 48.9, 40.6, 35.3, 24.5, 14.9; ^19^F NMR (470 MHz, CDCl_3_): δ 62.5; HRMS (ESI, positive) for C_16_H_18_F_3_NO_3_ (m/z): calculated 352.1131 (M + Na)^+^, found 352.1145.

#### Synthesis of (*R*)-*N*-hydroxy-2-((*S*)-3-methyl-2-oxo-3-(4-(trifluoromethyl)phenyl)pyrrolidin-1-yl)propanamide (**4**)

Hydroxylamine hydrochloride (234 mg, 34 mmol) in hot methanol (1.2 mL) was treated with a solution of KOH (281 mg, 50 mmol) in methanol (700 µL). The mixture was cooled to room temperature and the insoluble KCl was removed by filtration to yield a clear solution (approximately 1.76 M of NH_2_OH). The freshly prepared hydroxylamine solution (371 µL, 0.655 mmol) was added to a solution of 16 (43 mg, 0.131 mmol) in methanol (500 µL). The reaction mixture was stirred at room temperature for 25 min and then adjusted to pH 5–6 by addition of 1 N HCl while the flask was cooled on an ice-water bath. The precipitate was collected by filtration, rinsed with methanol/water (2:1, 500 µL), water (500 µL), and dried under vacuum to give amorphous compound 4 (39 mg, 90%). ^1^H NMR (500 MHz; CD_3_OD): δ 7.63 (d, *J* = 8.5 Hz, 2H), 7.59 (d, *J* = 8.5 Hz, 2H), 4.65 (q, *J* = 7.2 Hz, 1H), 3.61–3.52 (m, 2H), 2.43–2.39 (m, 1H), 2.25–2.20 (m, 1H), 1.55 (s, 3H), 1.43 (d, *J* = 7.2 Hz, 3H); ^13^C NMR (125 MHz; CD_3_OD): δ 179.3, 170.1, 149.6, 130.0 (q, *J* = 32.1 Hz), 128.2 (t, *J* = 14.4 Hz), 126.3 (q, *J* = 3.7 Hz), 125.7 (q, *J* = 269.4 Hz), 50.6, 50.0, 42.3, 36.8, 24.2, 15.2; ^19^F NMR (470 MHz, CD_3_OD): δ 64.0; HRMS (ESI, negative) for C_15_H_17_F_3_N_2_O_3_ (m/z): calculated 329.1108 (M-H)^-^, found 329.1109; HPLC purity 99.6%, retention time 7.36 min.

#### Synthesis of (*R*)-*N*-hydroxy-2-((*R*)-3-methyl-2-oxo-3-(4-(trifluoromethyl)phenyl)pyrrolidin-1-yl)propanamide (**4′**)

This compound was prepared from ester 16′ (47 mg, 0.413 mmol) using the procedure described for 4 in 72% yield as a white solid. m.p. 105–107 °C; ^1^H NMR (500 MHz; CD_3_OD): δ 7.64 (d, *J* = 8.5 Hz, 2H), 7.58 (d, *J* = 8.5 Hz, 2H), 4.63 (q, *J* = 7.2 Hz, 1H), 3.68–3.63 (m, 2H), 3.48–3.43 (m, 1H), 2.45–2.40 (m, 1H), 2.29–2.25 (m, 1H), 1.55 (s, 3H), 1.41 (d, *J* = 7.2 Hz, 3H); ^13^C NMR (125 MHz; CD_3_OD): δ 179.3, 170.1, 149.5, 130.0 (q, *J* = 32.3 Hz), 128.1, 126.4 (d, *J* = 3.8 Hz), 125.7 (q, *J* = 269.5 Hz), 50.6, 49.9, 42.3, 36.4, 24.2, 15.3; ^19^F NMR (470 MHz, CD_3_OD): δ 64.0; HRMS (ESI, negative) for C_15_H_17_F_3_N_2_O_3_ (m/z): calculated 329.1108 (M-H)^-^, found 329.1090; HPLC purity 96.01%, retention time 7.52 min.

#### Synthesis of methyl (2*R*)-2-(3-([1,1′-biphenyl]-4-yl)-3-methyl-2-oxopyrrolidin-1-yl)propanoate (**15**)

This compound was prepared from ester methyl 2-([1,1′-biphenyl]-4-yl)acetate (7) (601 mg, 2.76 mmol) using the procedure described above for 16 and afforded 156 mg of fast eluting isomer (15′), 118 mg of slow eluting desired isomer (15) (total yield 274 mg, 0.812 mmol, 29% for 4 steps) as a colorless oil.

**15:** ^1^H NMR (400 MHz; CDCl_3_): δ 7.58–7.55 (m, 4H), 7.49 (d, *J* = 6.8 Hz, 2H), 7.41 (d, *J* = 6.1 Hz, 2H), 7.31 (d, *J* = 6.1 Hz, 2H), 4.98 (q, *J* = 5.9 Hz, 1H), 3.67 (s, 3H), 3.42–3.35 (m, 2H), 2.50–2.45 (m, 1H), 2.19–2.13 (m, 1H), 1.59 (s, 3H), 1.46 (d, *J* = 5.9 Hz, 3H); ^13^C NMR (125 MHz; CDCl_3_): δ 177.8, 172.0, 142.7, 140.8, 139.5, 128.8, 127.2, 127.1, 127.0, 126.8, 52.2, 49.6, 48.6, 40.5, 35.9, 24.6, 14.8; HRMS (ESI, positive) for C_21_H_23_NO_3_ (m/z): calculated 360.1570 (M + Na)^+^, found 360.1572.

**15′:** ^1^H NMR (400 MHz; CDCl_3_): δ 7.58–7.55 (m, 4H), 7.47 (d, *J* = 6.8 Hz, 2H), 7.42 (d, *J* = 6.1 Hz, 2H), 7.33 (d, *J* = 6.1 Hz, 2H), 4.97 (q, *J* = 6.0 Hz, 1H), 3.74 (s, 3H), 3.50–3.46 (m, 1H), 3.36–3.31 (m, 1H), 2.49–2.44 (m, 1H), 2.27–2.21 (m, 1H), 1.60 (s, 3H), 1.45 (d, *J* = 6.0 Hz, 3H); ^13^C NMR (125 MHz; CDCl_3_): δ 177.9, 172.1, 142.8, 140.9, 139.7, 128.8, 127.3, 127.1, 126.6, 52.4, 49.7, 48.6, 40.6, 35.5, 24.7, 15.0; HRMS (ESI, positive) for C_21_H_23_NO_3_ (m/z): calculated 360.1570 (M + Na)^+^, found 360.1573.

#### Synthesis of (*R*)-2-((*S*)-3-([1,1′-biphenyl]-4-yl)-3-methyl-2-oxopyrrolidin-1-yl)-*N*-hydroxypropanamide (**3**)

This compound was prepared from ester 15 (58 mg, 0.172 mmol) using the procedure described above for compound 4 in 72% yield as a white solid: m.p. 160–162 °C; ^1^H NMR was the same as reported^18^; HRMS (ESI, negative) for C_20_H_22_N_2_O_3_ (m/z): calculated 337.1547 (M-H)^-^, found 337.1547; HPLC purity 99.4%, retention time 7.96 min.

#### Synthesis of (*R*)-2-((*R*)-3-([1,1′-biphenyl]-4-yl)-3-methyl-2-oxopyrrolidin-1-yl)-*N*-hydroxypropanamide (**3′**)

This compound was prepared from ester 15′ (46 mg, 0.136 mmol) using the procedure described above for compound 4 in 98% yield as a white solid: m.p. 137–138 °C; ^1^H NMR (500 MHz; CD_3_OD): δ 7.59–7.57 (m, 4H), 7.43–7.39 (m, 4H), 7.31 (t, *J* = 7.4 Hz, 1H), 4.66 (q, *J* = 7.2 Hz, 1H), 3.65–3.60 (m, 1H), 3.44–3.35 (m, 1H), 2.45–2.40 (m, 1H), 2.22–2.17 (m, 1H), 1.54 (s, 3H), 1.39 (d, *J* = 7.2 Hz, 3H); ^13^C NMR (125 MHz; CD_3_OD): δ 180.0, 170.1, 143.9, 141.8, 141.0, 129.9, 128.4, 128.1, 127.8, 127.7, 50.3, 49.8, 42.3, 36.7, 24.4, 15.3; HRMS (ESI, negative) for C_20_H_22_N_2_O_3_ (m/z): calculated 337.1547 (M-H)^-^, found 337.1541; HPLC purity 99.20%, retention time 7.91 min.

#### Synthesis of methyl (*R*)-2-((*S*)-3-methyl-2-oxo-3-phenylpyrrolidin-1-yl)propanoate (**17**)

This compound was prepared from ester methyl 2-phenylacetate (9) (561 µL, 4.00 mmol) using the procedure described above for 16 and afforded 224 mg of fast eluting isomer, 98 mg of slow eluting desired isomer (17), and 190 mg of mixture containing a mixture of both isomers (total yield 512 mg, 1.96 mmol, 49% for 4 steps) as a colorless oil. ^1^H NMR (400 MHz; CDCl_3_): δ 7.42 (d, *J* = 7.2 Hz, 2H), 7.33 (d, *J* = 7.6 Hz, 2H), 7.23 (t, *J* = 7.2 Hz, 1H), 4.98 (q, *J* = 7.4 Hz, 1H), 3.68 (s, 3H), 3.37 (t, *J* = 6.7 Hz, 2H), 2.49–2.43 (m, 1H), 2.18–2.11 (m, 1H), 1.56 (s, 3H), 1.46 (d, *J* = 7.4 Hz, 3H); ^13^C NMR (125 MHz; CDCl_3_): δ 177.9, 171.9, 143.7, 128.5, 126.8, 126.4, 52.3, 49.7, 48.9, 40.5, 36.1, 24.8, 14.9; HRMS (ESI, positive) for C_15_H_19_NO_3_ (m/z): calculated 284.1257 (M + Na)^+^, found 284.1259.

#### Synthesis of (*R*)-*N*-hydroxy-2-((*S*)-3-methyl-2-oxo-3-phenylpyrrolidin-1-yl)propanamide (**5**)

This compound was prepared from ester 17 (65 mg, 0.249 mmol) using the procedure described above for compound 4 in 49% yield as amorphous material. The structure was determined by comparison with authentic samples prepared by the literature procedure^18^; HPLC purity 96.1%, retention time 6.17 min.

#### Synthesis of methyl (*R*)-2-((*S*)-3-methyl-2-oxo-3-(3-(trifluoromethyl)phenyl)pyrrolidin-1-yl)propanoate (**18**)

This compound was prepared from methyl 2-(3-(trifluoromethyl)phenyl)acetate (10) (898 mg, 4.12 mmol) using the procedure described above for 16 afforded 21 mg of fast eluting isomer, 96 mg of slow eluting desired isomer (18), and 278 mg of mixture containing a mixture of both isomers (total yield 396 mg, 1.12 mmol, 29% for 4 steps) as a colorless oil. ^1^H NMR (400 MHz; CDCl_3_): δ 7.67–7.64 (m, 2H), 7.50–7.46 (m, 2H), 4.98 (q, *J* = 7.4 Hz, 1H), 3.67 (s, 3H), 3.40–3.35 (m, 2H), 2.48–2.42 (m, 1H), 2.24–2.17 (m, 1H), 1.58 (s, 3H), 1.48 (d, *J* = 7.4 Hz, 3H); ^13^C NMR (125 MHz; CDCl_3_): δ 177.1, 171.7, 144.7, 130.7 (q, *J* = 31.8 Hz), 130.0, 128.9, 123.7 (q, *J* = 3.8 Hz), 123.6 (q, *J* = 3.8 Hz), 124.2 (q, *J* = 270.8 Hz), 52.3, 49.6, 48.8, 40.4, 35.7, 24.7, 14.7; ^19^F NMR (470 MHz, CDCl_3_): δ 62.4; HRMS (ESI, positive) for C_16_H_18_F_3_NO_3_ (m/z): calculated 352.1131 (M + Na)^+^, found 352.1135.

#### Synthesis of (*R*)-*N*-hydroxy-2-((*S*)-3-methyl-2-oxo-3-(3-(trifluoromethyl)phenyl)pyrrolidin-1-yl)propanamide (**6**)

This compound was prepared from ester 18 (67 mg, 0.204 mmol) using the procedure described above for compound 4 in 43% yield as amorphous material. ^1^H NMR (500 MHz; CD_3_OD): δ 7.74 (s, 1H), 7.66 (d, *J* = 7.2 Hz, 1H), 7.56–7.51 (m, 2H), 4.64 (q, *J* = 7.2 Hz, 1H), 3.62–3.53 (m, 2H), 2.44–2.39 (m, 1H), 2.27–2.12 (m, 1H), 1.55 (s, 3H), 1.43 (d, *J* = 7.2 Hz, 3H); ^13^C NMR (125 MHz; CD_3_OD): δ 179.3, 170.0, 146.5, 131.7 (q, *J* = 31.0 Hz), 131.4, 130.3, 124.6 (q, *J* = 3.8 Hz), 124.1 (q, *J* = 3.8 Hz), 124.2 (q, *J* = 272.6 Hz), 50.3, 50.0, 42.3, 40.4, 36.6, 24.3, 15.2; ^19^F NMR (470 MHz, CD_3_OD): δ 64.0; HRMS (ESI, negative) for C_15_H_17_F_3_N_2_O_3_ (m/z): calculated 329.1108 (M–H)^−^, found 329.1117 ; HPLC purity 95.5%, retention time 7.91 min.

### Biology

MMP-1 inhibitory assay was performed using the assay kit according to the manufacturer’s protocol. A 96-well clear microplate (1/2 volume), 30.6 U/µL of MMP-1 enzyme, MMP-1 substrate (25 mM in DMSO), and colorimetric assay buffer were contained in MMP1 Inhibitor Screening Assay Kit (ab139443, abcam). 2-[(2-Methylpropyl)[(4-methoxyphenyl)sulfonyl]amino]acetohydroximic acid (NNGH) was used as a positive control of an MMP-1 inhibitor. The MMP-1 substrate and DMSO solution of compound were thawed at room temperature. A compound (10 mM in DMSO) was diluted at 1/200 in assay buffer and brought to 37 °C. Also, the MMP-1 substrate was diluted at 1/200 in assay buffer and brought to 37 °C. The MMP-1 enzyme was diluted at 1/25 in assay buffer and warmed up to 37 °C as soon as the assay was started. After the assay buffer was pipetted appropriately into each well, 20 µL of the prepared MMP-1 enzyme solution and the desired concentration of test inhibitor solutions (20 µL; final concentrations: 0.1–100 µM) were added to the wells. The microplate was incubated for 30 min at 37 °C. The 10 µL of the prepared MMP-1 substrate solution was added into each well to allow the reaction start. The absorbance of the wells was measured at A_412nm_ using a microplate reader every 1 min, and data analysis was performed.

### Pharmacophore fitting

To predict binding interaction between compounds and MMP-1, a pharmacophore model was constructed from the crystal structure of SC44463/MMP-1 (PDB: 1FBL) using LigandScout 4.4 (InteLigand GmbH). Then, three pharmacophore features of SC44463 were used including a hydrogen bond acceptor (HA), hydrophobic (Hy) moiety, and zinc binding location feature (ZL). For pharmacophore evaluation, the scoring function was set to ‘Relative Pharmacophore-Fit’. For all other parameters, default values were used. Compounds were fit to the SC44463 pharmacophore model followed by interaction energy minimization with MMP-1.

## Supplementary information

Supplementary information.
