# Supplementary material for: Prediction of an MMP-1 inhibitor activity cliff using the SAR matrix approach and its experimental validation
Source: Sci Rep. 2020 Sep 7;10:14710. doi: 10.1038/s41598-020-71696-2 (PMC7477548; doi:10.1038/s41598-020-71696-2)

Supporting Information

Prediction of an MMP-1 Inhibitor Activity Cliff Using the SAR Matrix Approach and Its Experimental Validation

Yasunobu Asawa,[a],[b] Atsushi Yoshimori,[c] Jürgen Bajorath,*[d] Hiroyuki Nakamura*[a]

[a] Laboratory for Chemistry and Life Science, Institute of Innovative Research, Tokyo Institute of Technology, Nagatsuta-cho, Midori-ku, Yokohama 226-8503, Japan

[b] School of Life Science and Technology, Tokyo Institute of Technology, Nagatsuta-cho, Midori-ku, Yokohama 226-8503, Japan

[c] Institute for Theoretical Medicine, Inc., Fujisawa, Kanagawa 251-8555, Japan

[d] Department of Life Science Informatics, B-IT, LIMES Program Unit Chemical Biology and Medicinal Chemistry, Rheinische Friedrich-Wilhelms-Universität, Endenicher Allee 19c, Bonn D-53115, Germany

List of contents

1. Figure S1. Pharmacophore fitting	S2
Figure S2. The distance of hydrogen bond	S3
2. NMR spectra and HRMS spectrum of compounds	
Compound **3**	S4-S5
Compound **3**’	S5-S6
Compound **4**	S7-S8
Compound **4’**	S9-S10
Compound **5**	S11-S12
Compound **6**	S12-S13
Compound **15**	S14-S15
Compound **15’**	S16-S17
Compound **16**	S17-S19
Compound **16’**	S19-S21
Compound **17**	S21-S22
Compound **18**	S23-S24
3. Purity analysis of tested compounds **3-6** by HPLC	S25-S26

(a) (b) (c)

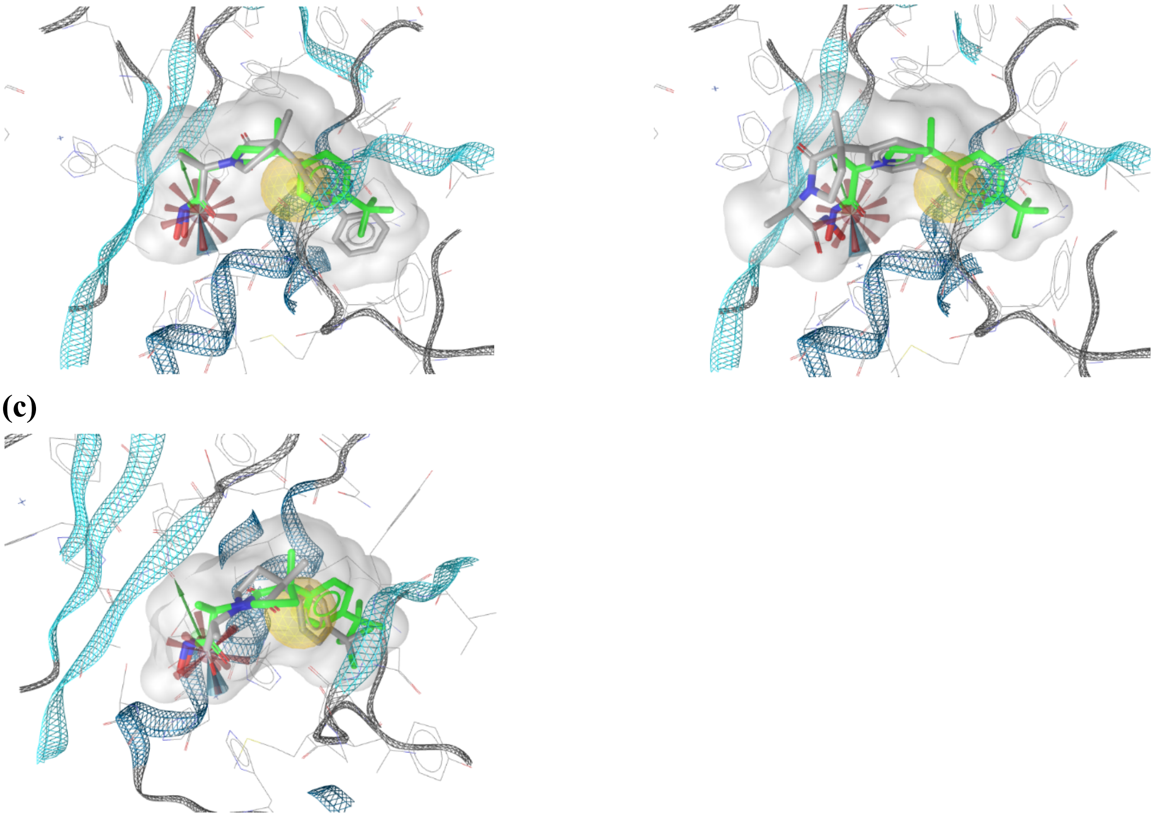

Figure S1. (a) Pharmacophore fitting of compound **3** (black: **3**; green: **4**); (b) conformation of **3** refined by interaction energy optimization; Binding energy; (c) Pharmacophore fitting of compound **4’** (black: **4’**; green: **4**).

(a) **(b)**

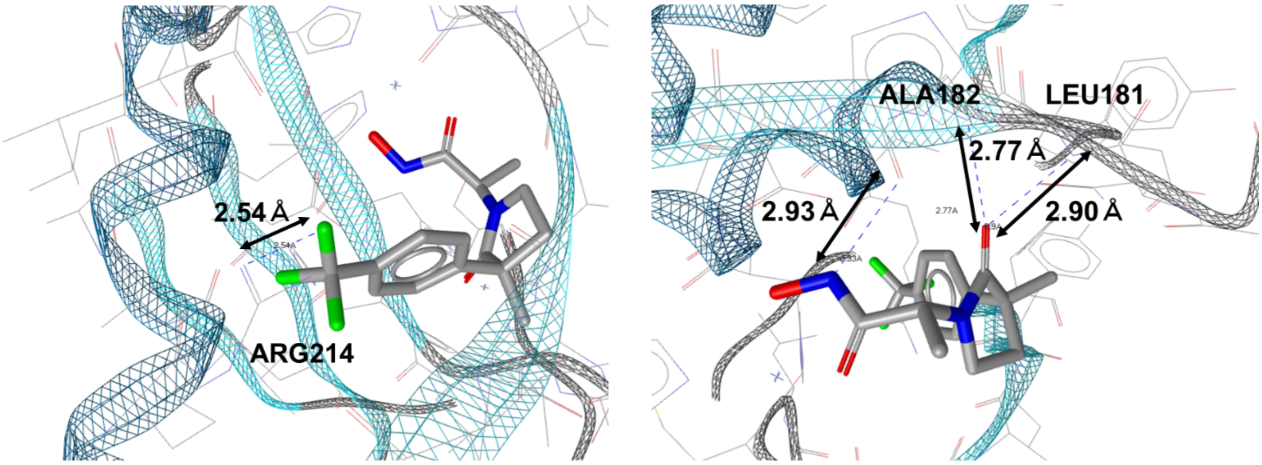

Figure S2. (a) The calculated distance between C-F bond of the (*R*,*S*)-enantiomer of compound **4** and N-H bond of ARG214; (b) The calculated distance between hydroxamic acid moiety of compound 4 and carbonyl group of ALA182. In addition, the calculated distance between γ-lactam C=O bond of compound 4 and N-H bond of LEU181 and ALA182.

2. NMR spectra and HRMS spectrum of Compounds

(*R*)-2-((*S*)-3-([1,1'-Biphenyl]-4-yl)-3-methyl-2-oxopyrrolidin-1-yl)-*N*-hydroxypropanamide (3)

^1H NMR ($CDCl_3$, 500 MHz)

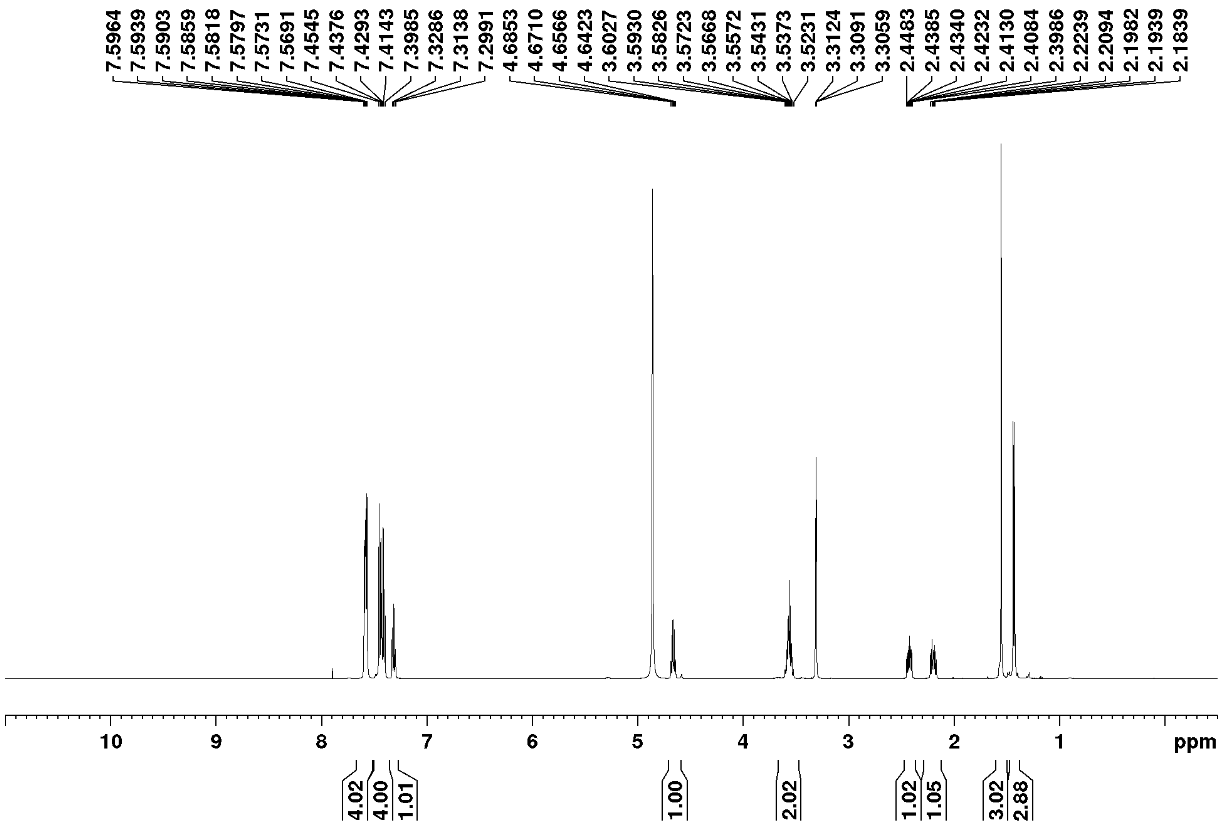

^{13}C NMR ($CDCl_3$, 125 MHz)

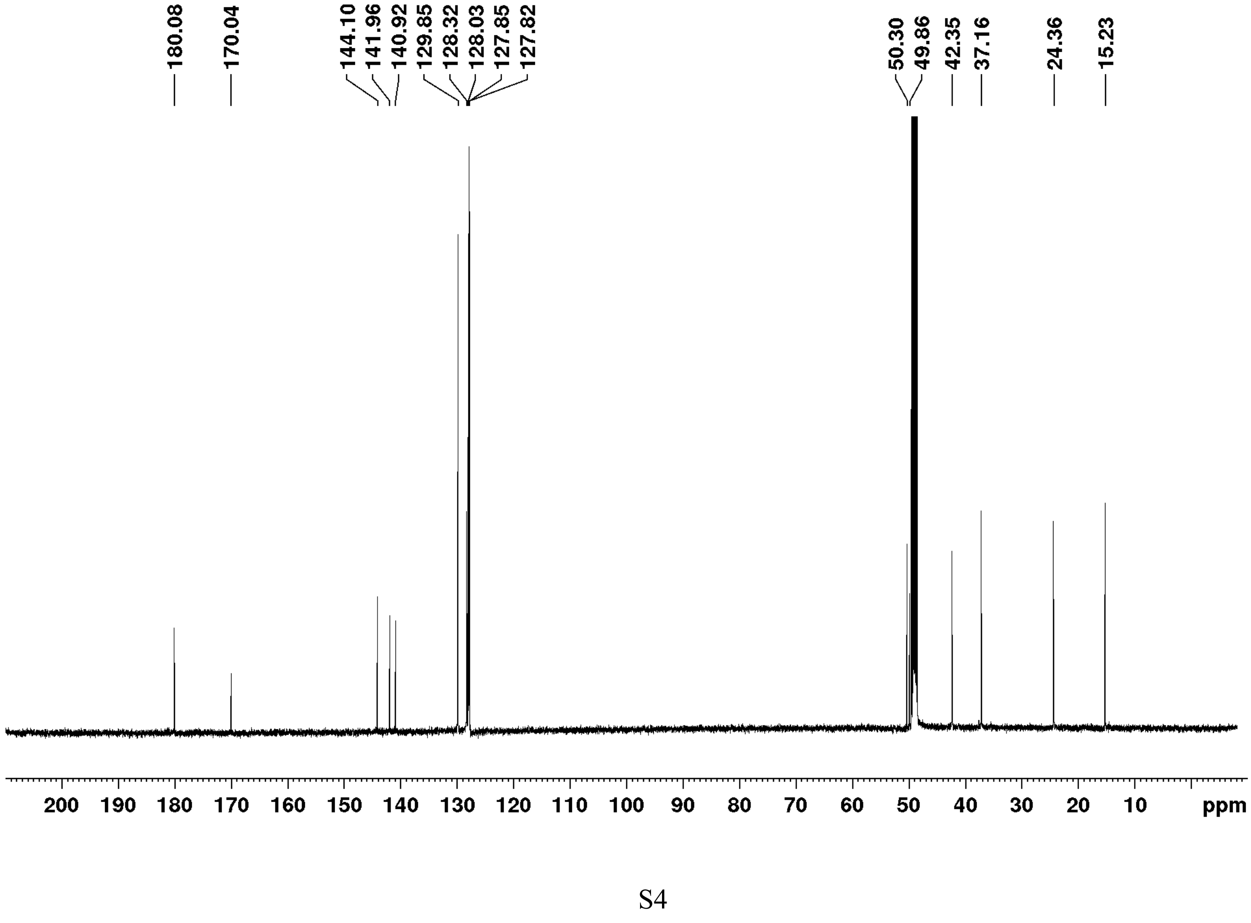

HRMS (ESI, negative) for $C_{20}H_{22}N_2O_3$ (m/z): calculated 337.1547 (M-H)$^-$, found 337.1547

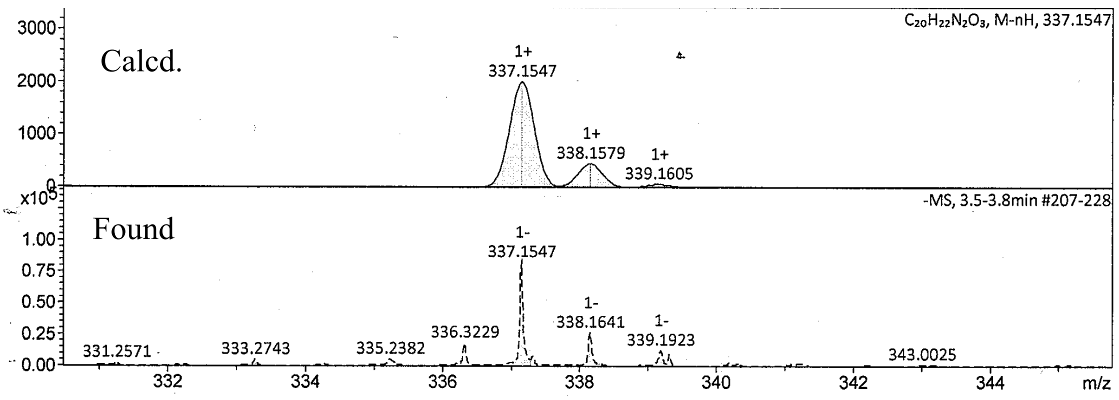

(*R*)-2-((*R*)-3-([1,1'-Biphenyl]-4-yl)-3-methyl-2-oxopyrrolidin-1-yl)-*N*-hydroxypropanamide (3')

^{1}H NMR ($CDCl_3$, 500 MHz)

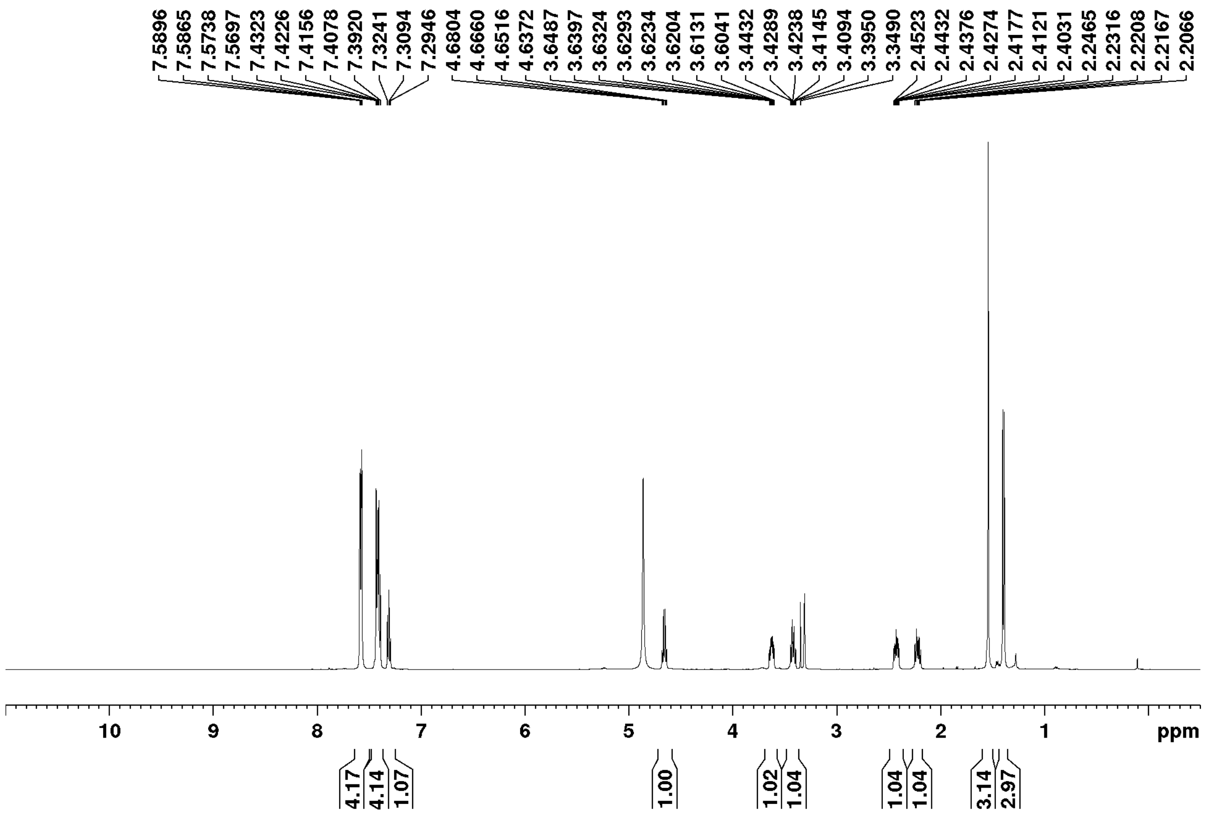

^{13}C NMR ($CDCl_3$, 125 MHz)

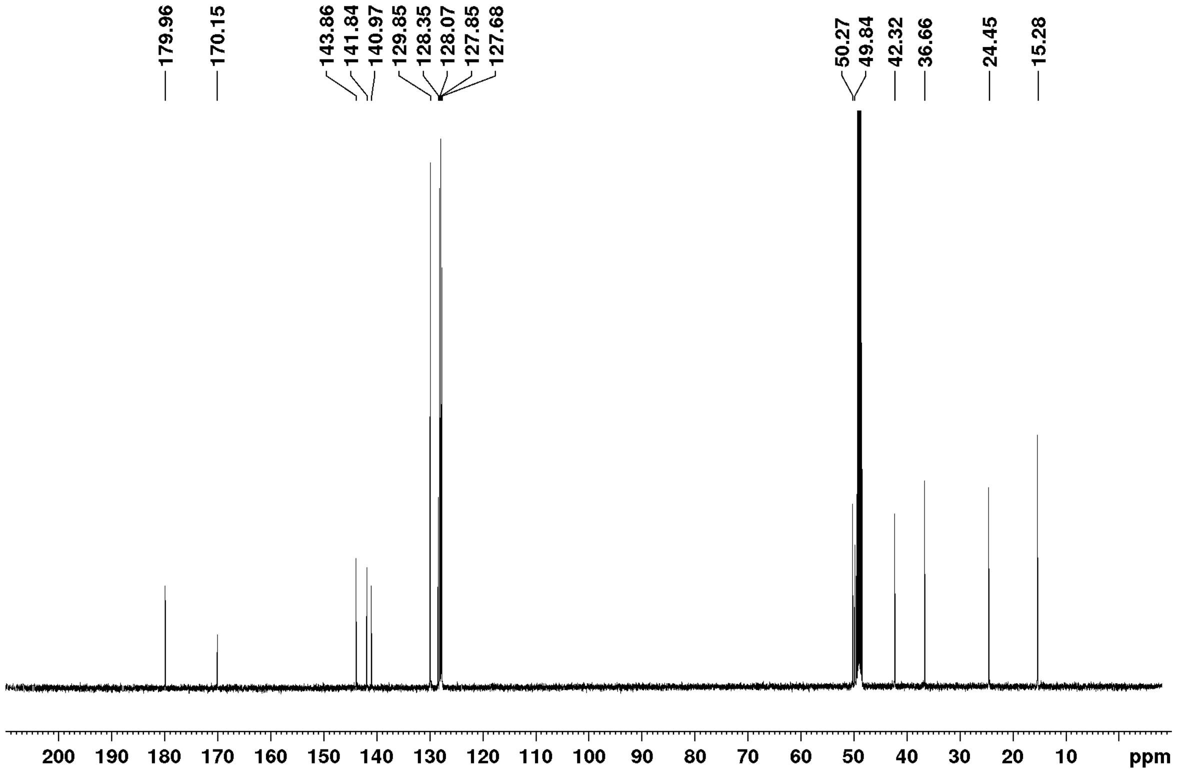

HRMS (ESI, negative) for $C_{20}H_{22}N_2O_3$ (m/z): calculated 337.1547 (M-H)$^-$, found 337.1541

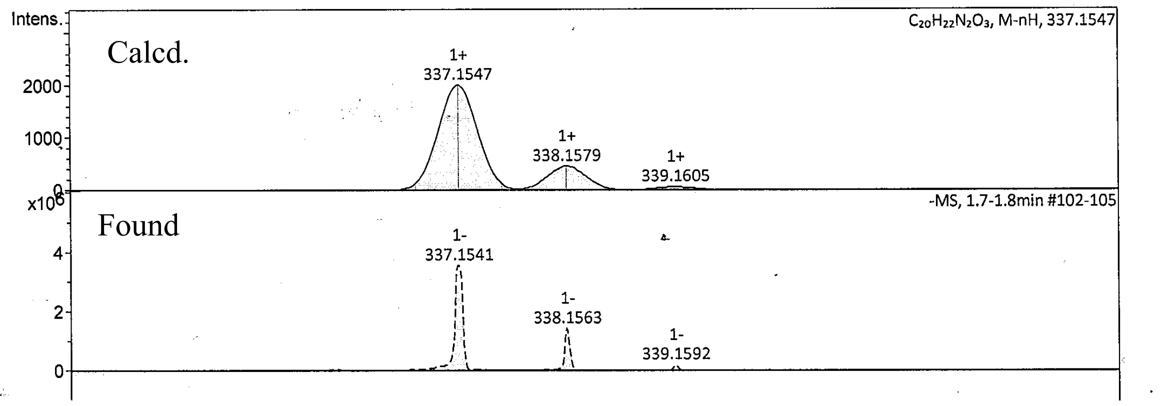

(*R*)-*N*-Hydroxy-2-((*S*)-3-methyl-2-oxo-3-(4-(trifluoromethyl)phenyl)pyrrolidin-1-yl)propanamide (4)

^{1}H NMR ($CDCl_3$, 500 MHz)

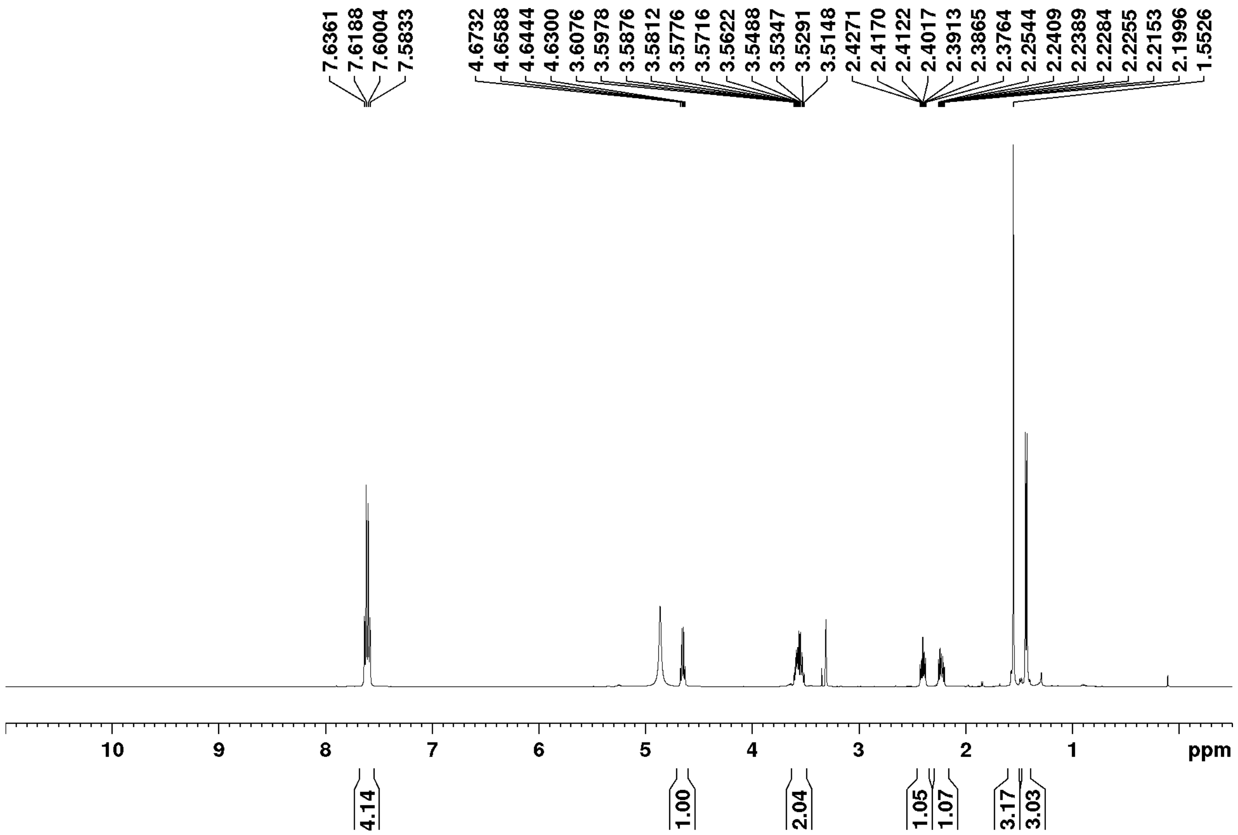

^{13}C NMR ($CDCl_3$, 125 MHz)

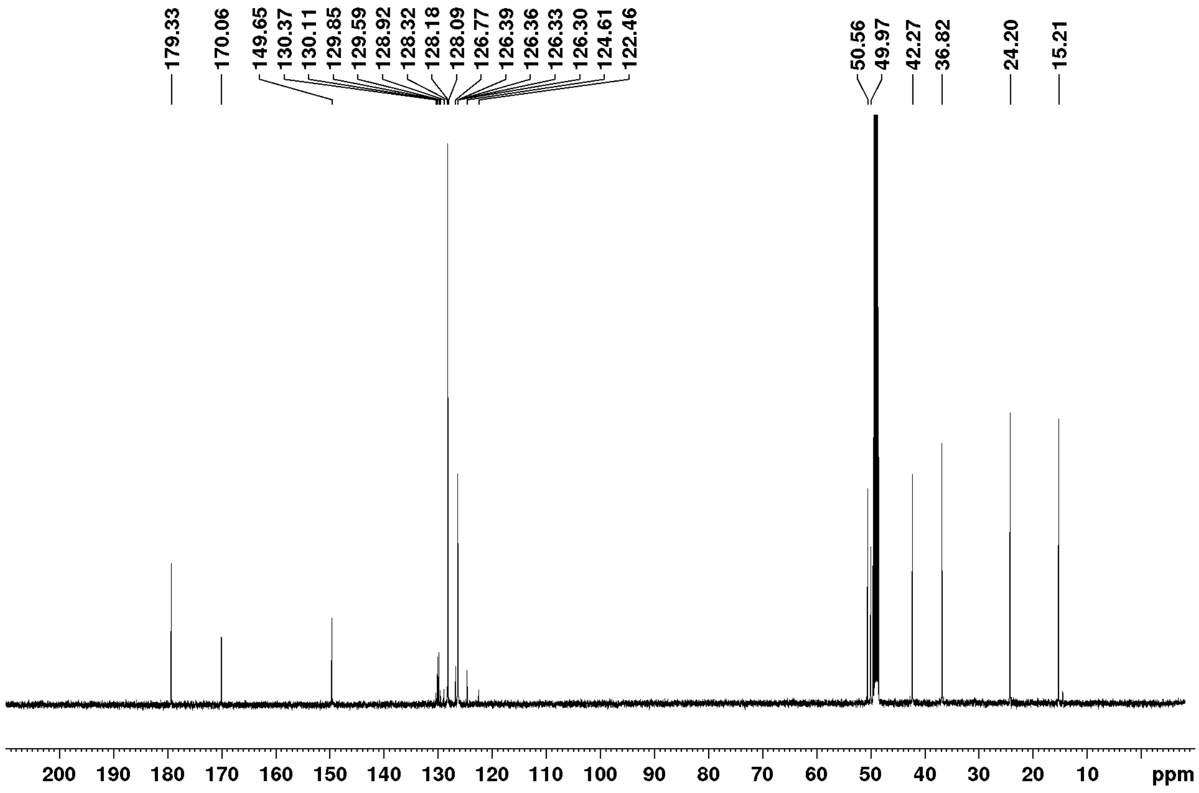

^{19}F NMR ($CDCl_3$, 470 MHz)

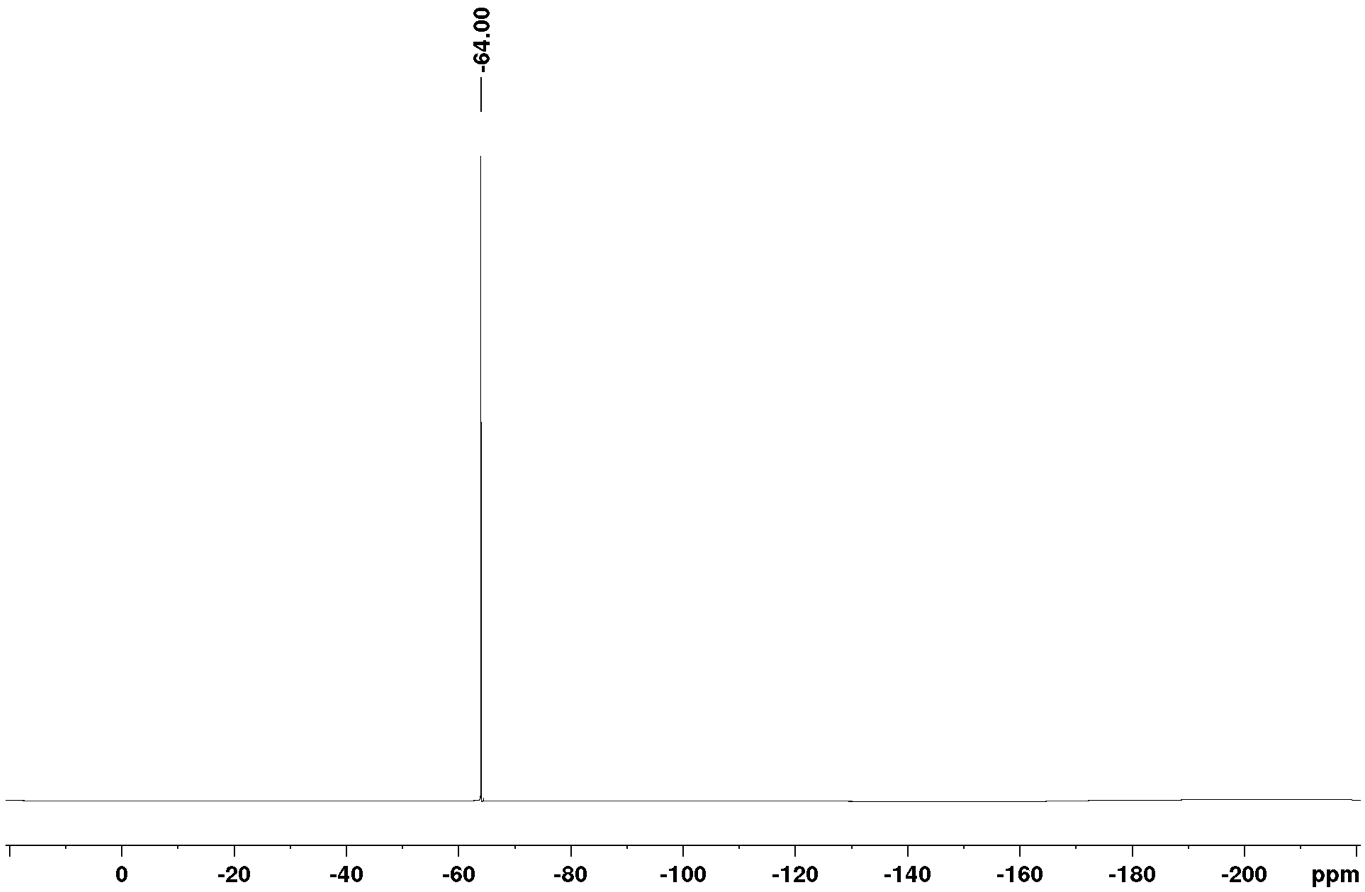

HRMS (ESI, negative) for $C_{15}H_{17}F_3N_2O_3$ (m/z): calculated 329.1108 $(M-H)^-$, found 329.1109

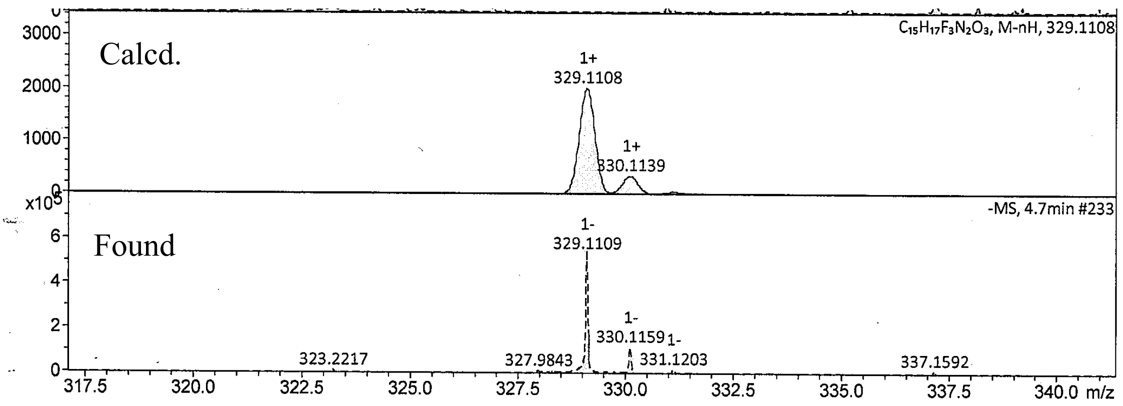

(*R*)-*N*-Hydroxy-2-((*R*)-3-methyl-2-oxo-3-(4-(trifluoromethyl)phenyl)pyrrolidin-1-yl)propanamide (4')

^{1}H NMR ($CDCl_3$, 500 MHz)

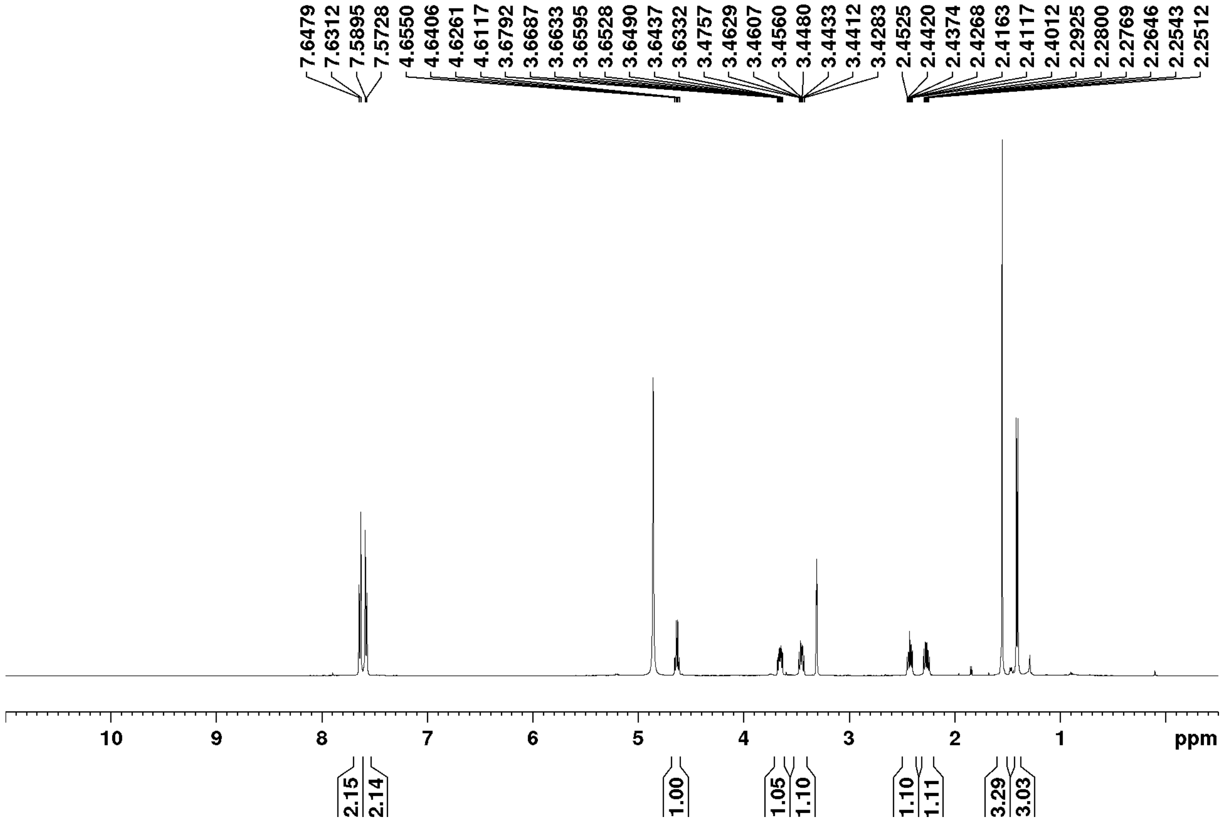

^{13}C NMR ($CDCl_3$, 125 MHz)

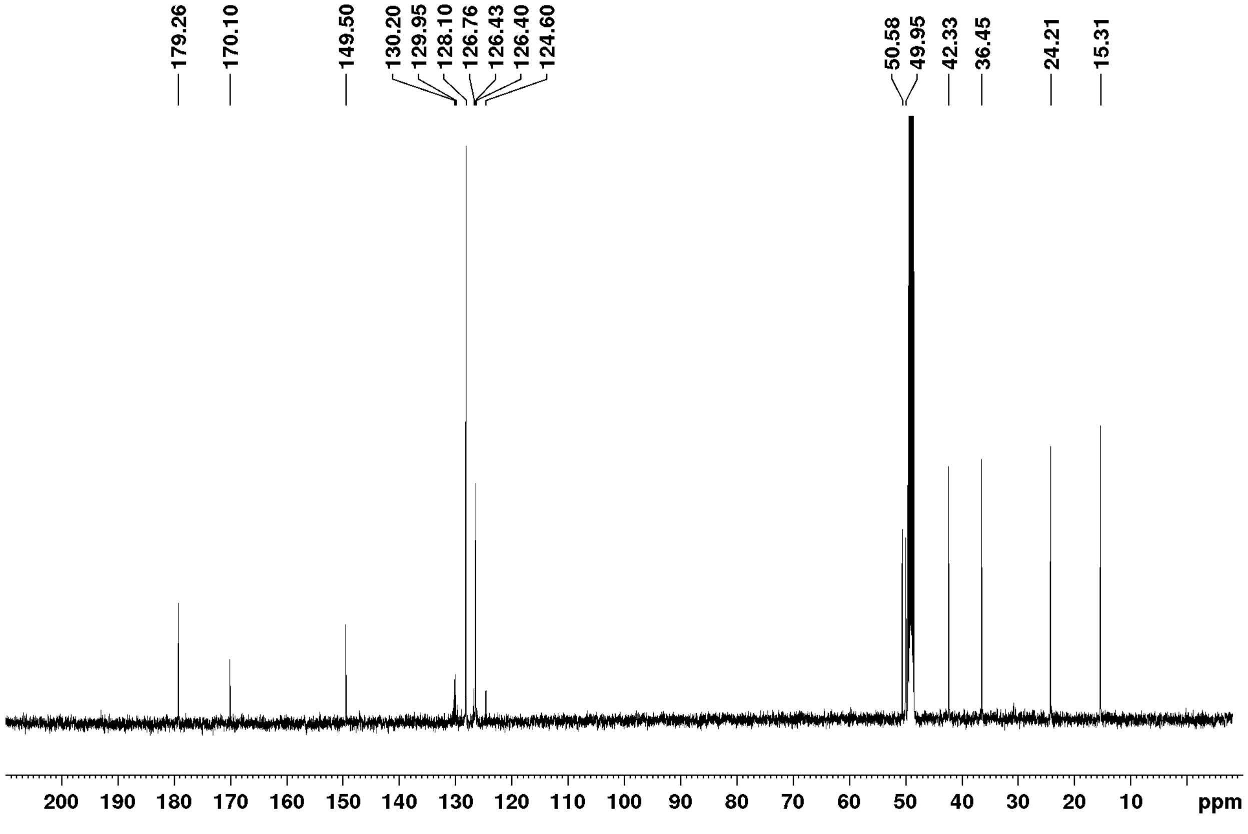

^{19}F NMR ($CDCl_3$, 470 MHz)

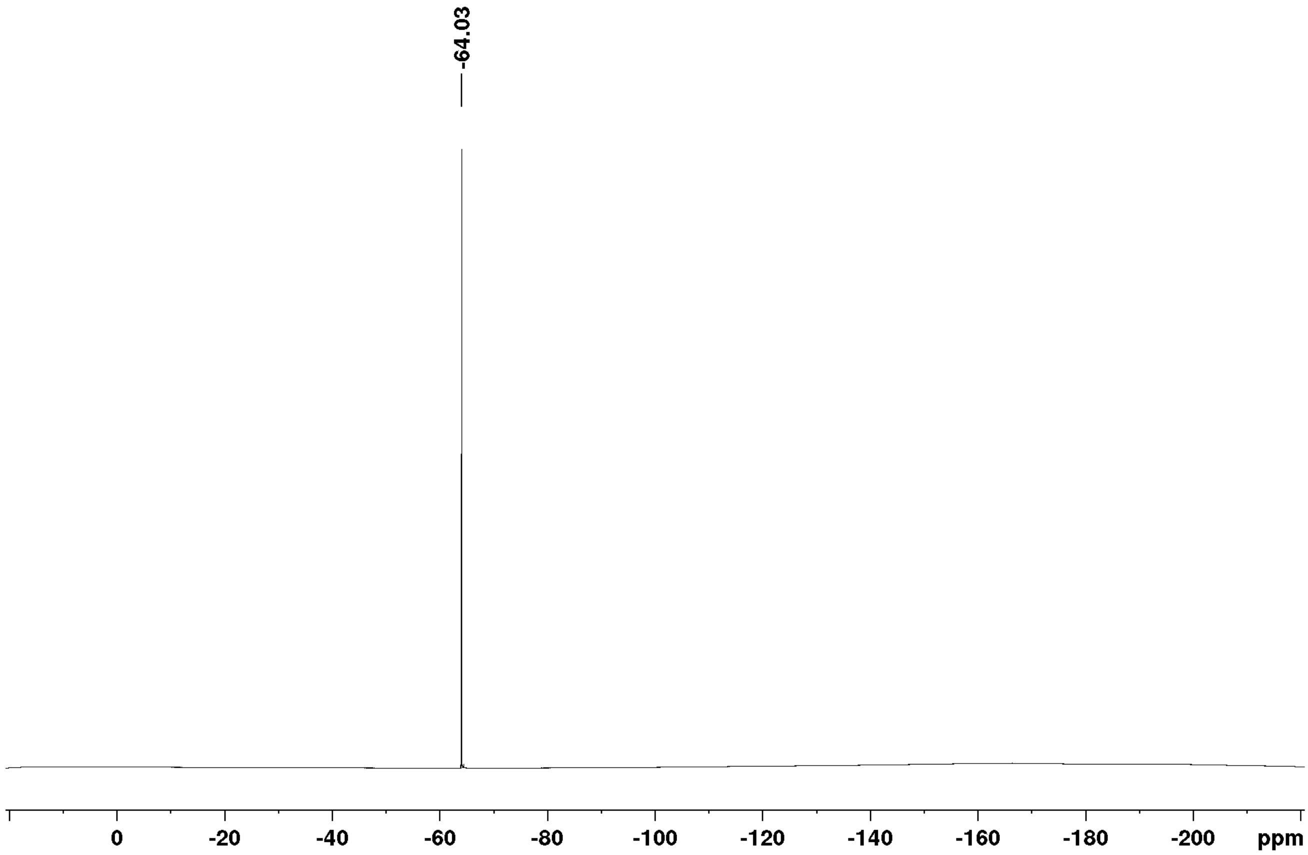

HRMS (ESI, negative) for $C_{15}H_{17}F_3N_2O_3$ (m/z): calculated 329.1108 $(M-H)^-$, found 329.1090

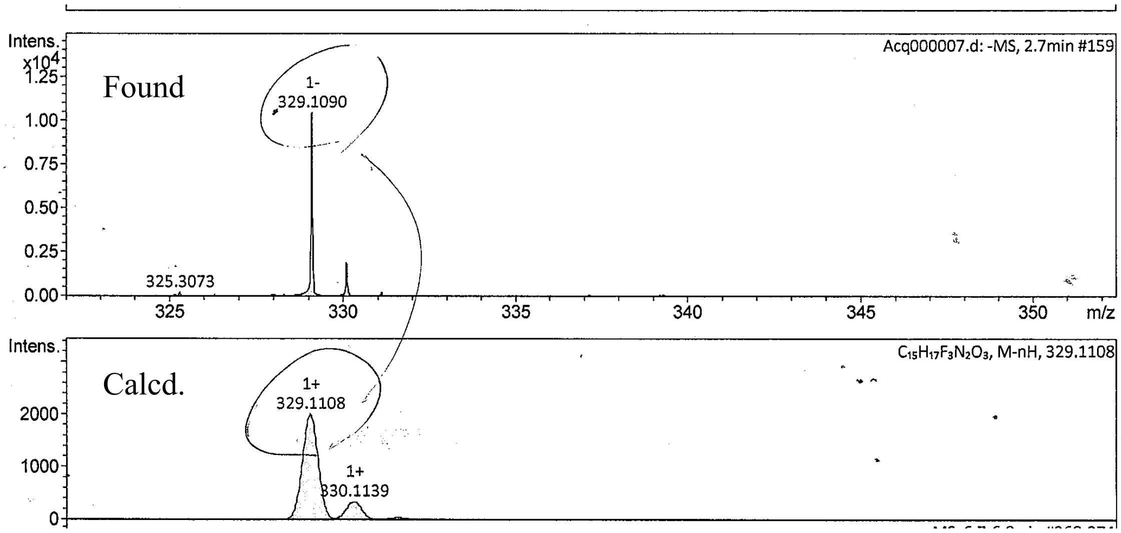

(*R*)-*N*-Hydroxy-2-((*S*)-3-methyl-2-oxo-3-phenylpyrrolidin-1-yl)propanamide (5)

^{1}H NMR ($CDCl_3$, 500 MHz)

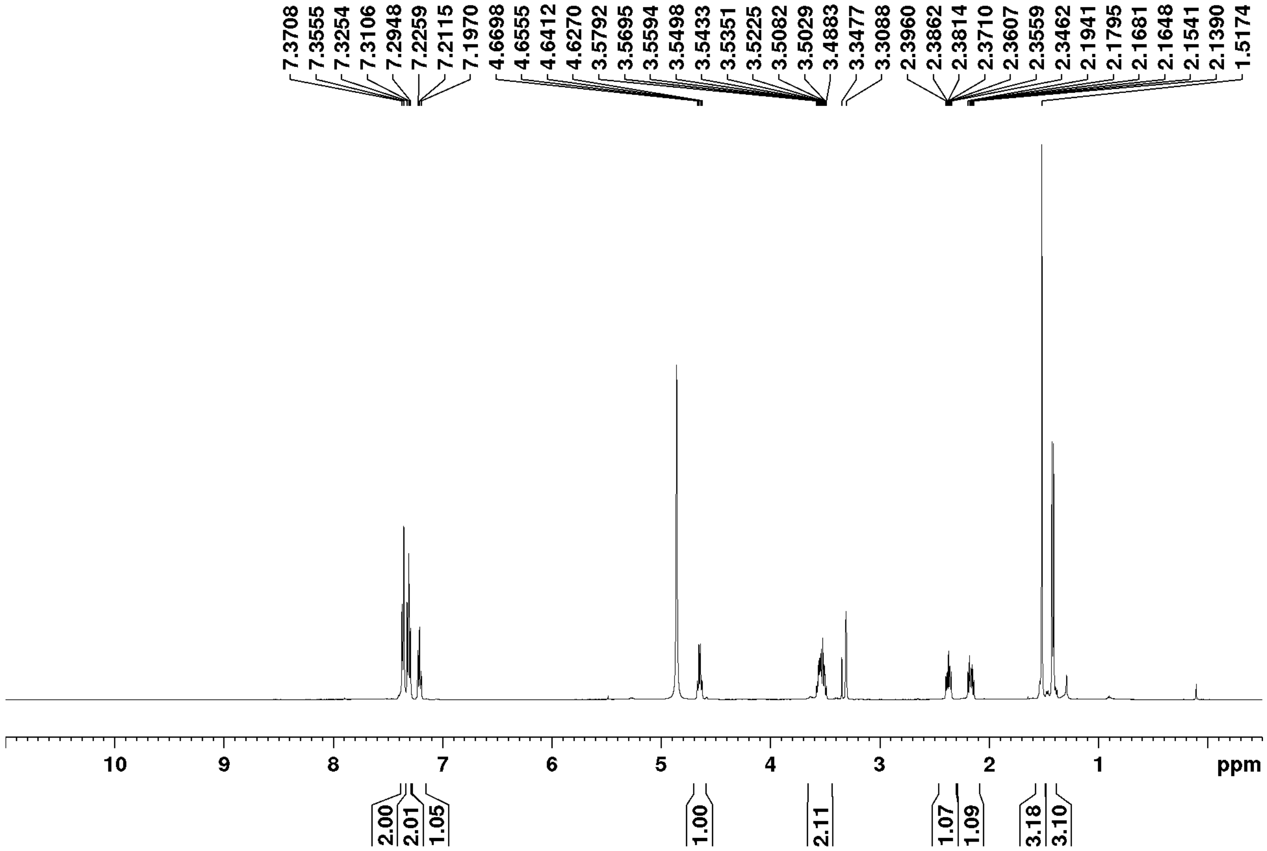

^{13}C NMR ($CDCl_3$, 125 MHz)

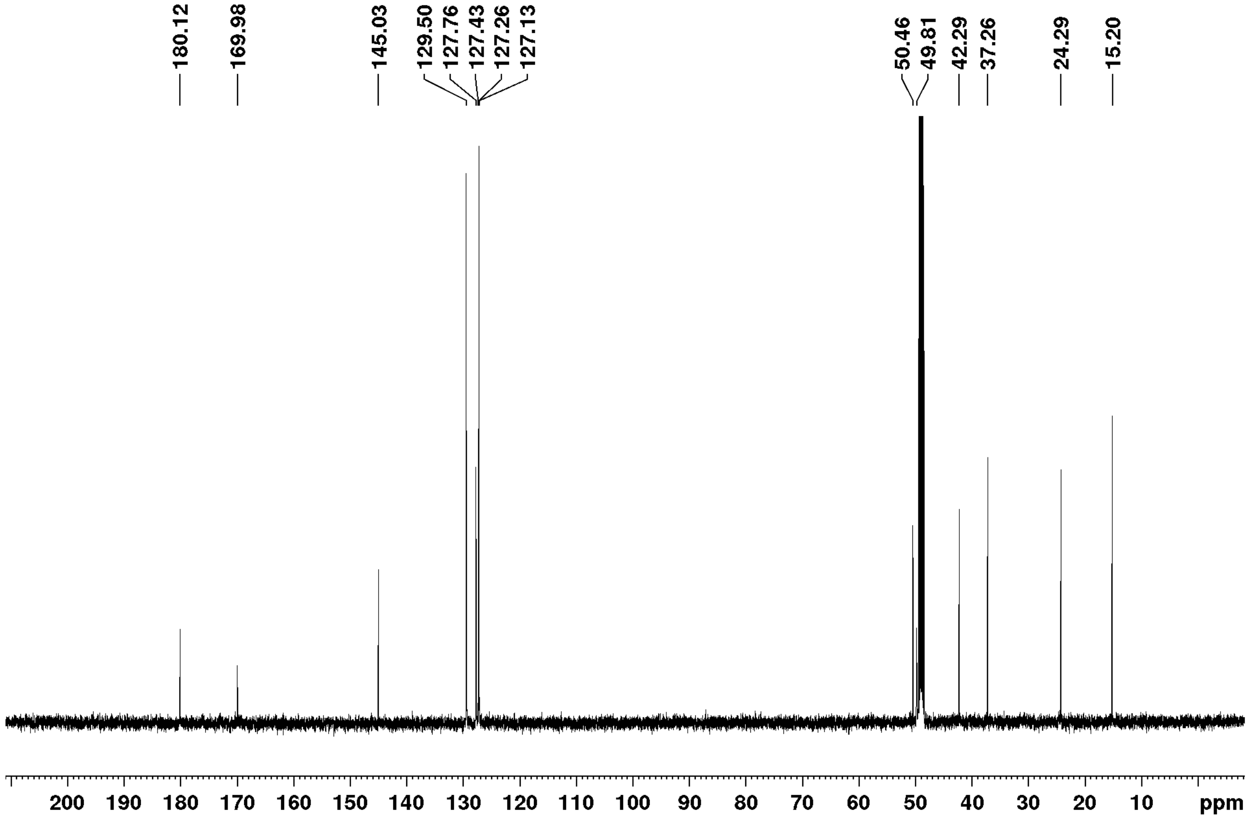

HRMS (ESI, positive) for $C_{16}H_{18}F_3NO_3$ (m/z): calculated 285.1210 $(M+Na)^+$, found 285.1213

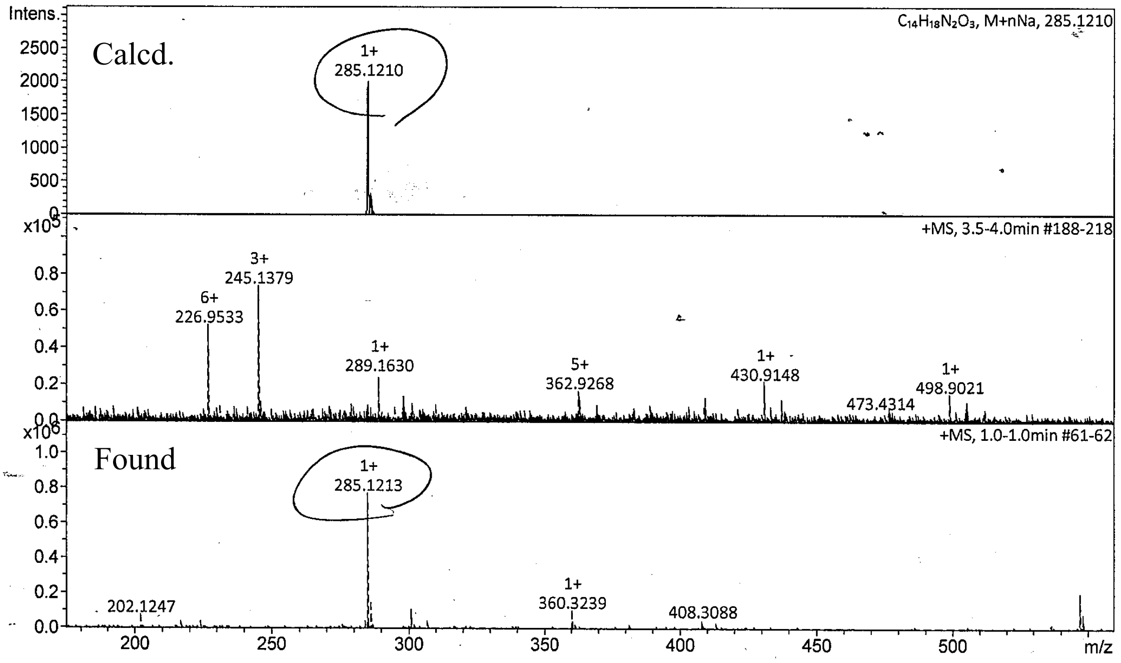

(*R*)-*N*-Hydroxy-2-((*S*)-3-methyl-2-oxo-3-(3-(trifluoromethyl)phenyl)pyrrolidin-1-yl)propanamide (6)

^{1}H NMR ($CDCl_3$, 500 MHz)

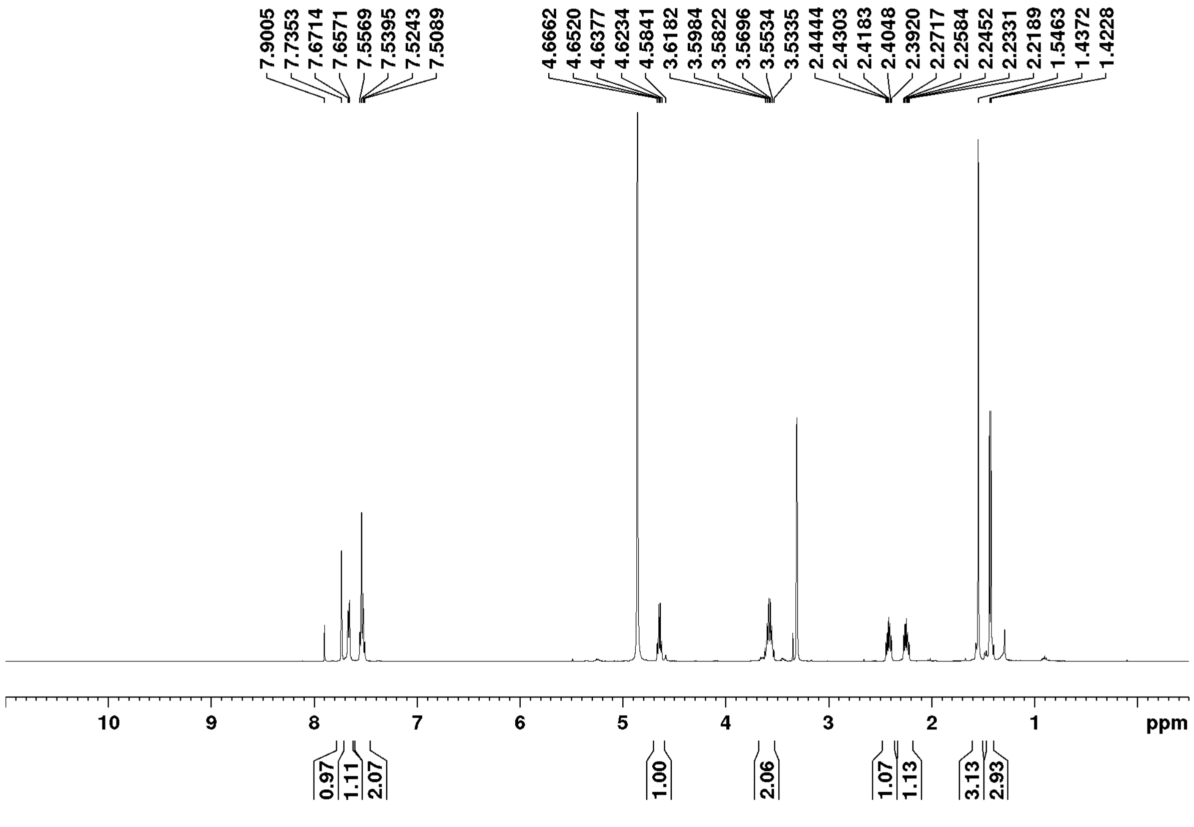

^{13}C NMR ($CDCl_3$, 125 MHz)

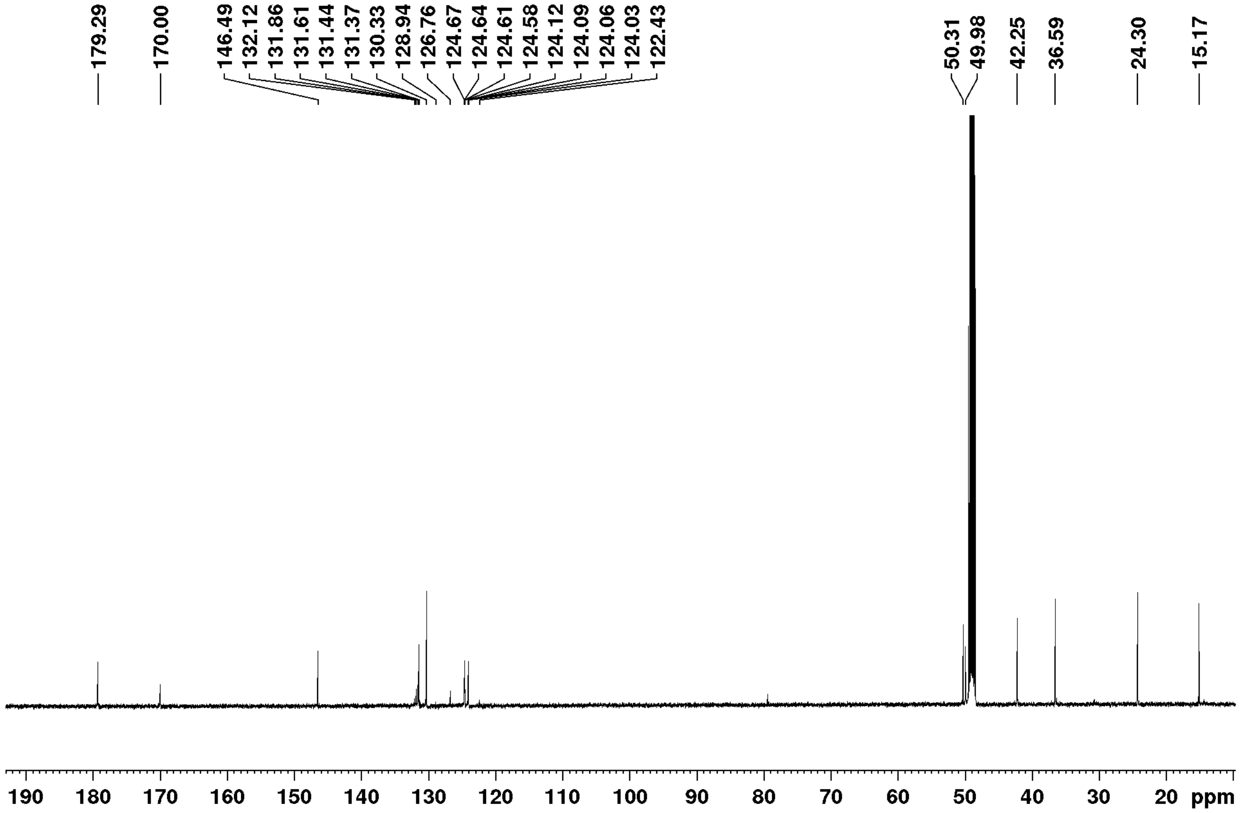

^{19}F NMR ($CDCl_3$, 470 MHz)

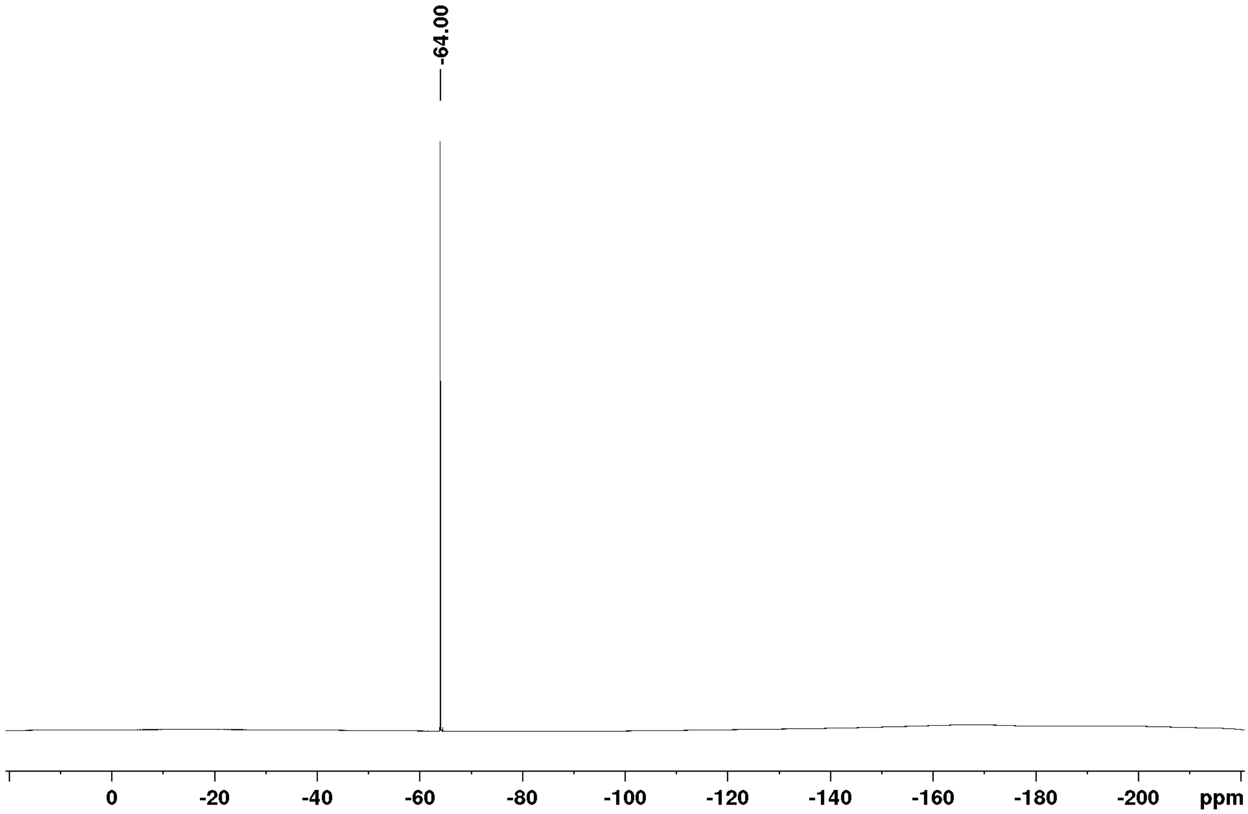

HRMS (ESI, negative) for $C_{15}H_{17}F_3N_2O_3$ (m/z): calculated 329.1108 (M-H)$^-$, found 329.1117

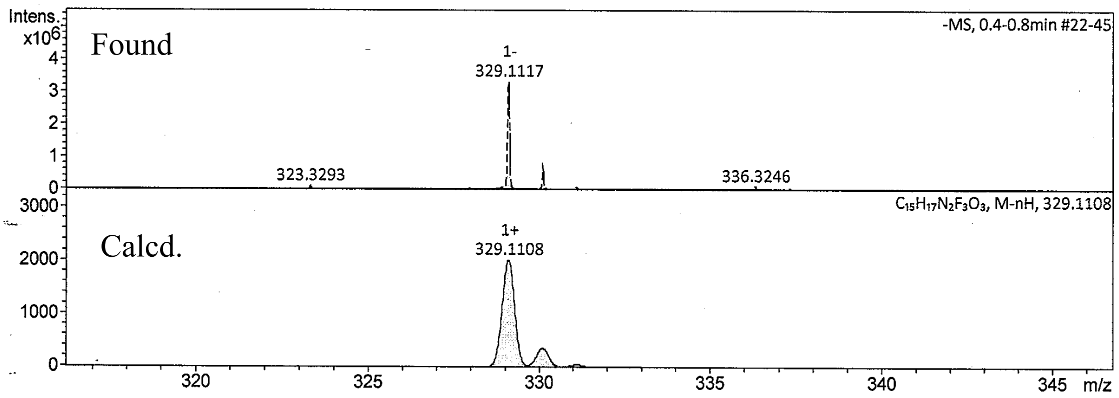

Methyl (*R*)-2-((*S*)-3-([1,1'-biphenyl]-4-yl)-3-methyl-2-oxopyrrolidin-1-yl)propanoate (15)

^{1}H NMR ($CDCl_3$, 400 MHz)

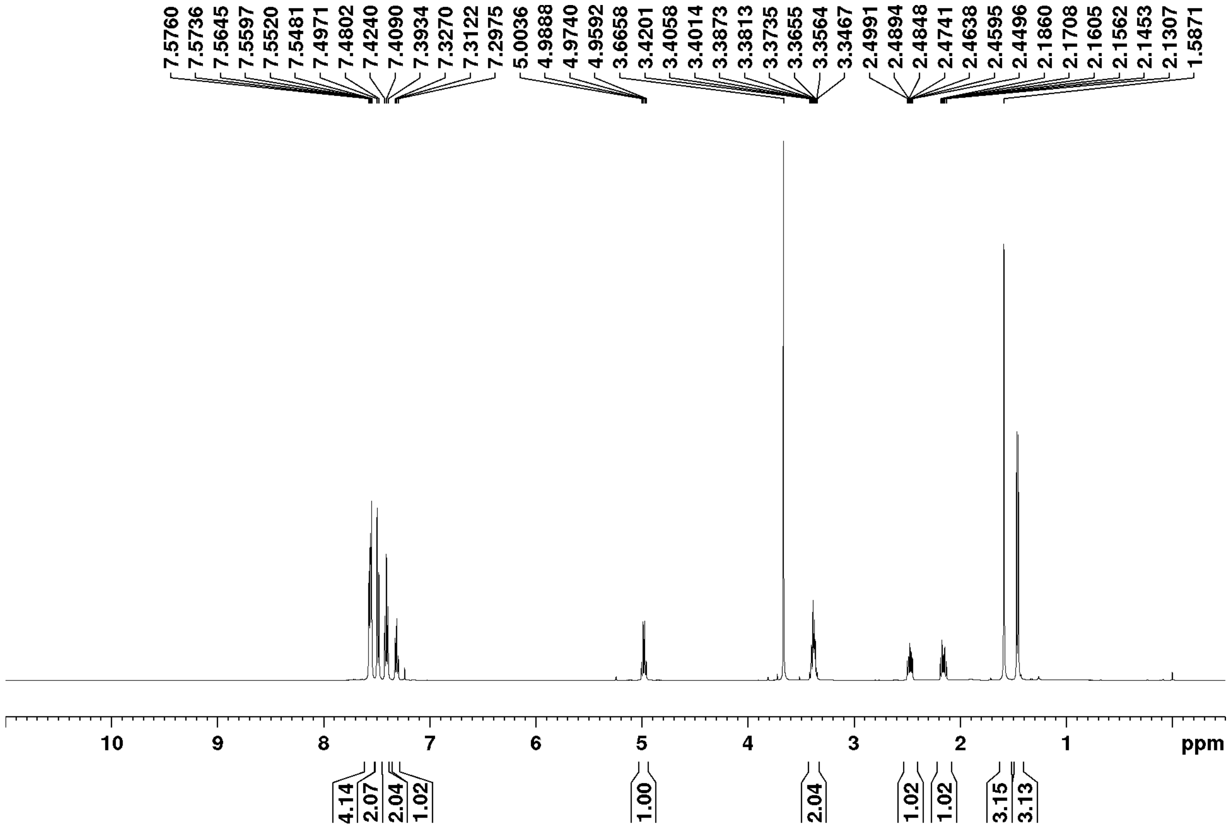

^{13}C NMR ($CDCl_3$, 125 MHz)

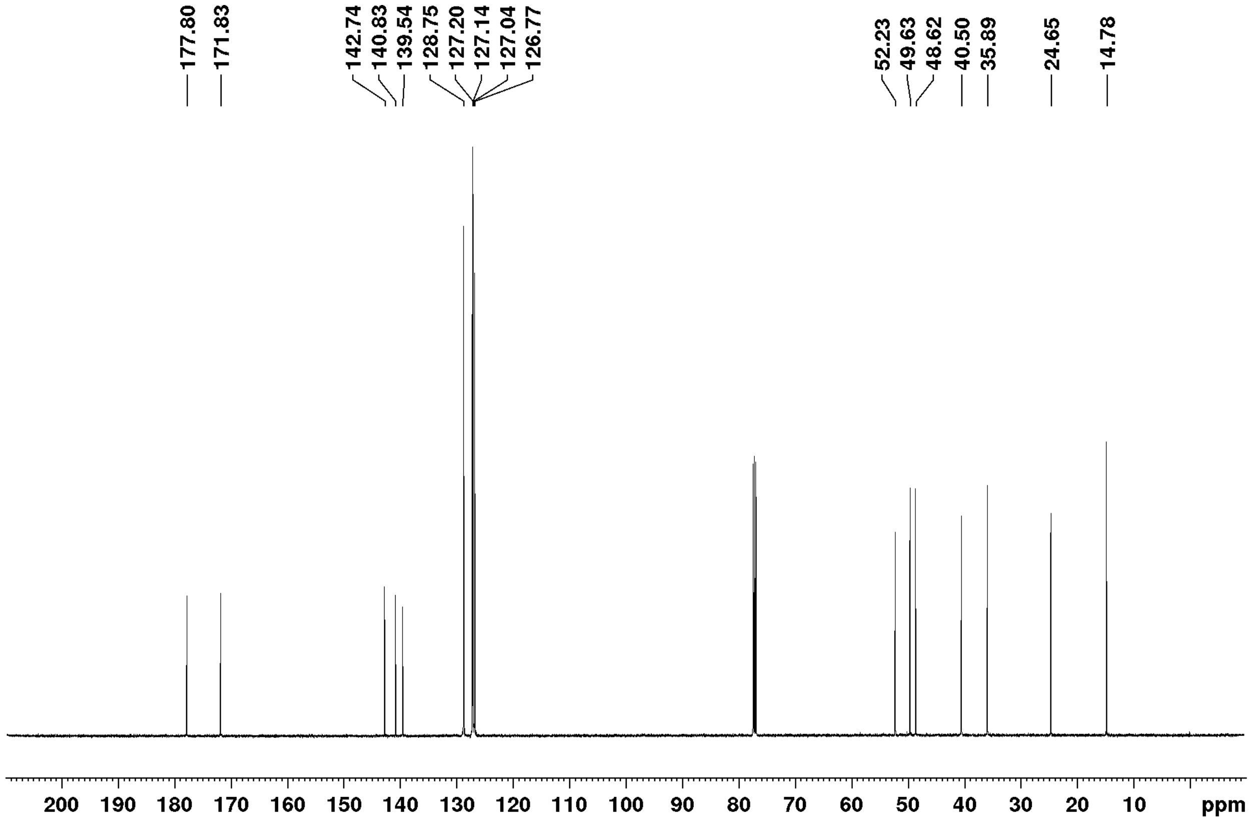

HRMS (ESI, positive) for $C_{21}H_{23}NO_3$ (m/z): calculated 360.1570 $(M+Na)^+$, found 360.1572

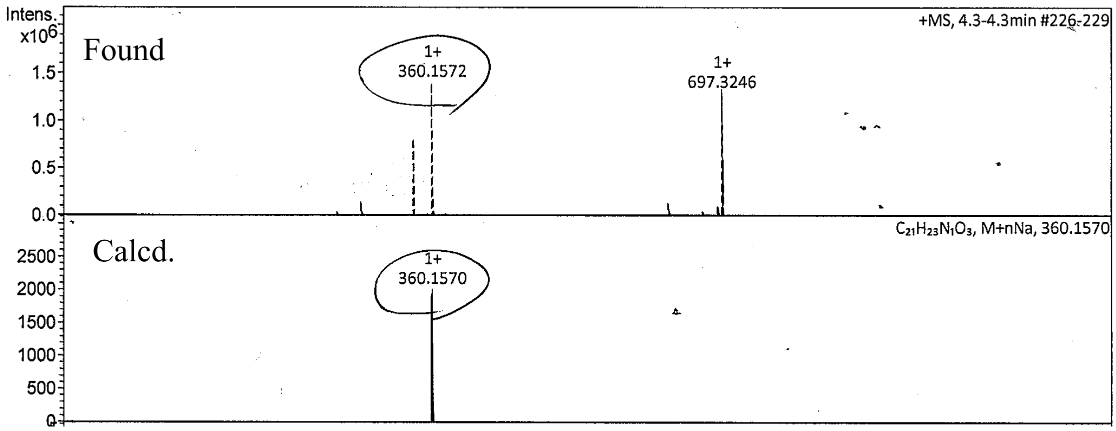

Methyl (*R*)-2-((*R*)-3-([1,1'-biphenyl]-4-yl)-3-methyl-2-oxopyrrolidin-1-yl)propanoate (15')

^{1}H NMR ($CDCl_3$, 400 MHz)

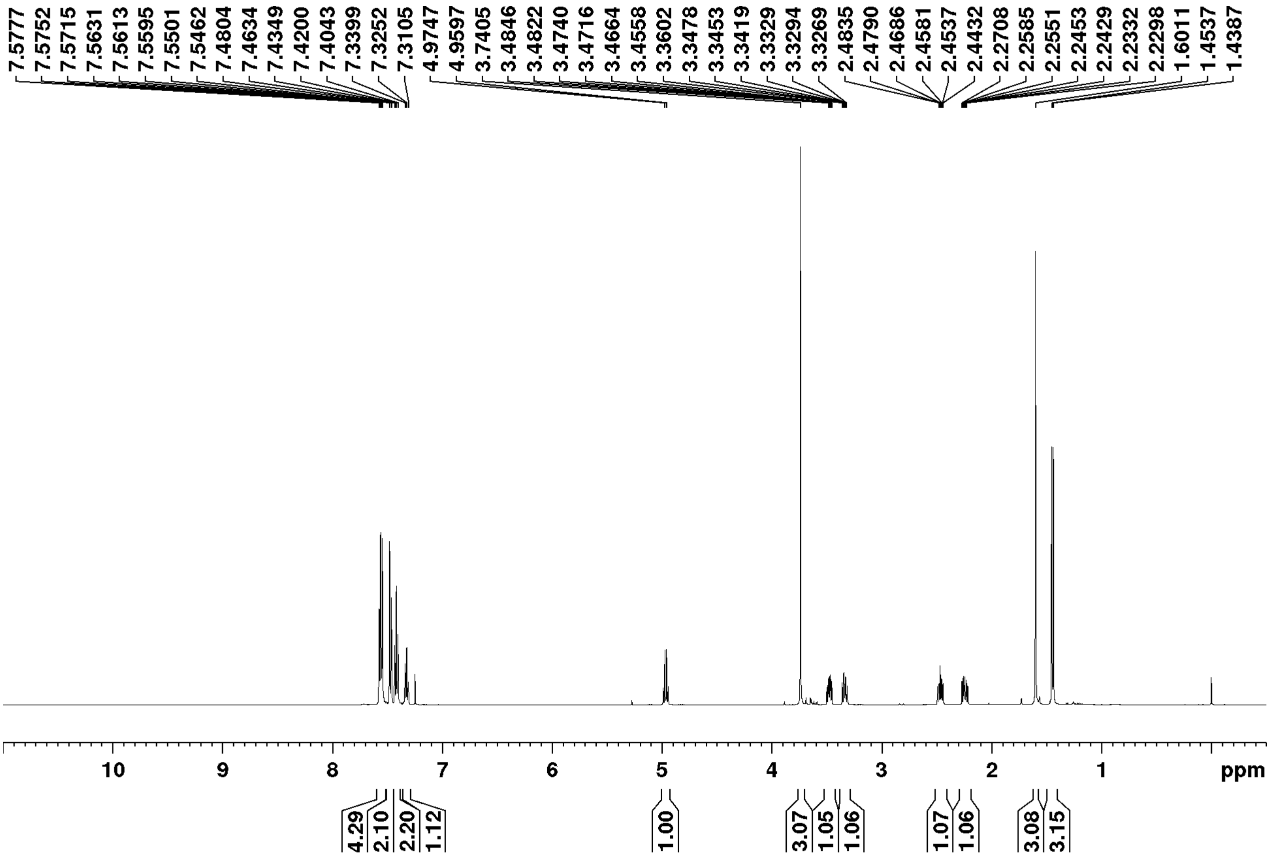

^{13}C NMR ($CDCl_3$, 125 MHz)

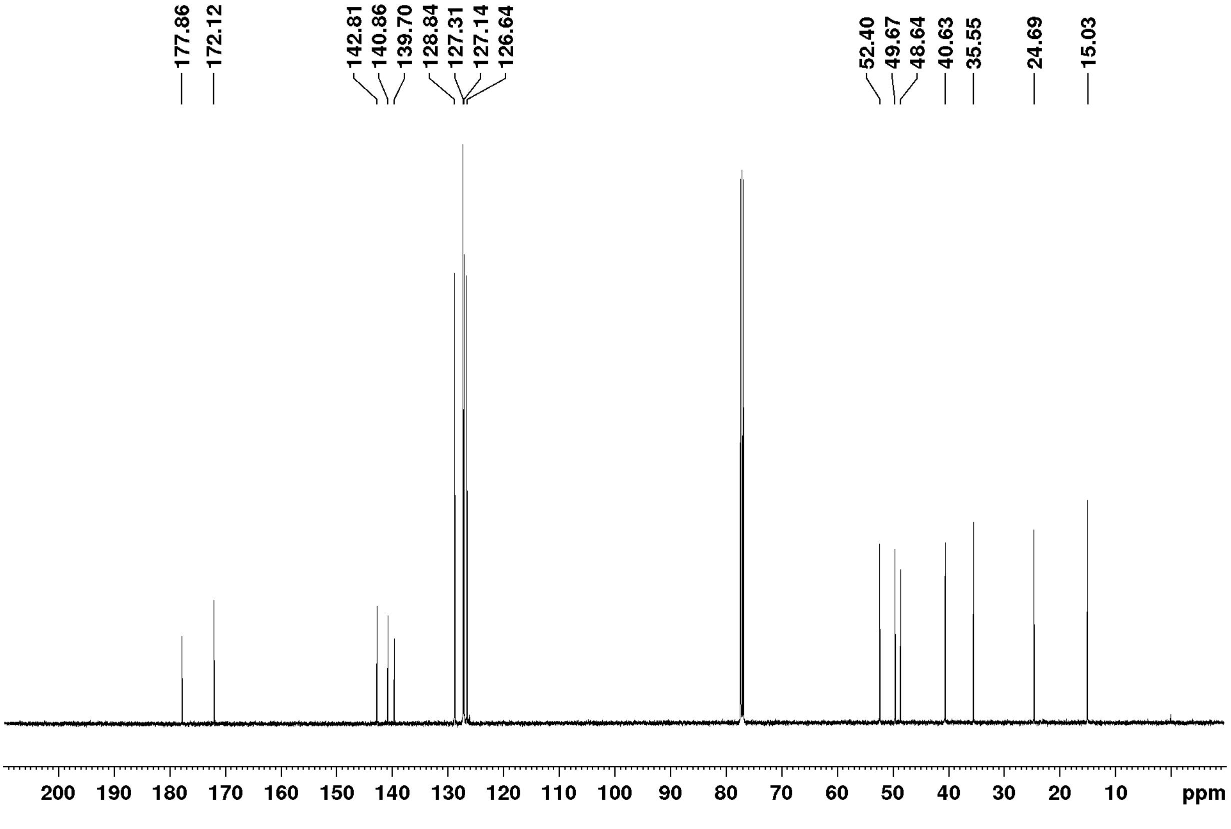

HRMS (ESI, positive) for $C_{21}H_{23}NO_3$ (m/z): calculated 360.1570 $(M+Na)^+$, found 360.1573

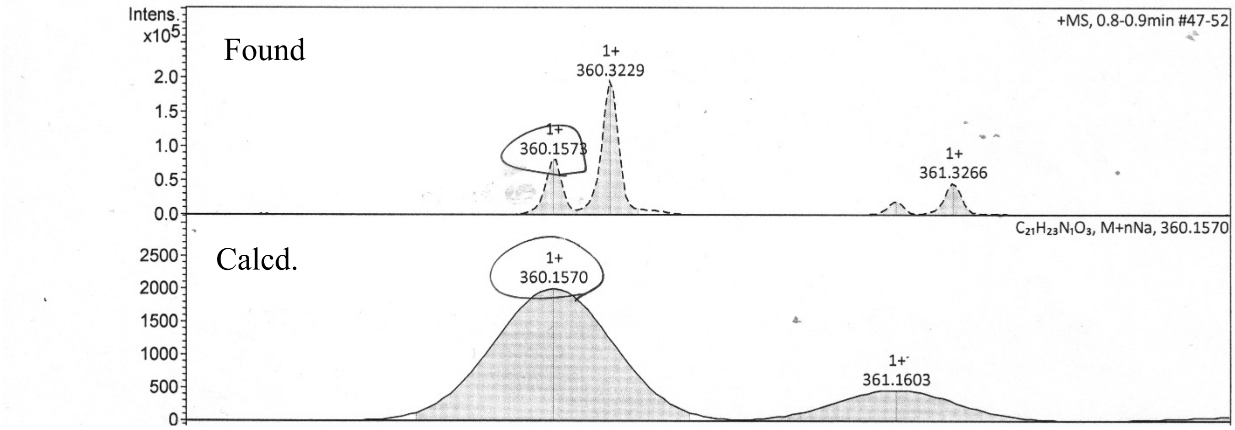

Methyl (*R*)-2-((*S*)-3-methyl-2-oxo-3-(4-(trifluoromethyl)phenyl)pyrrolidin-1-yl)propanoate (16)

^{1}H NMR ($CDCl_3$, 400 MHz)

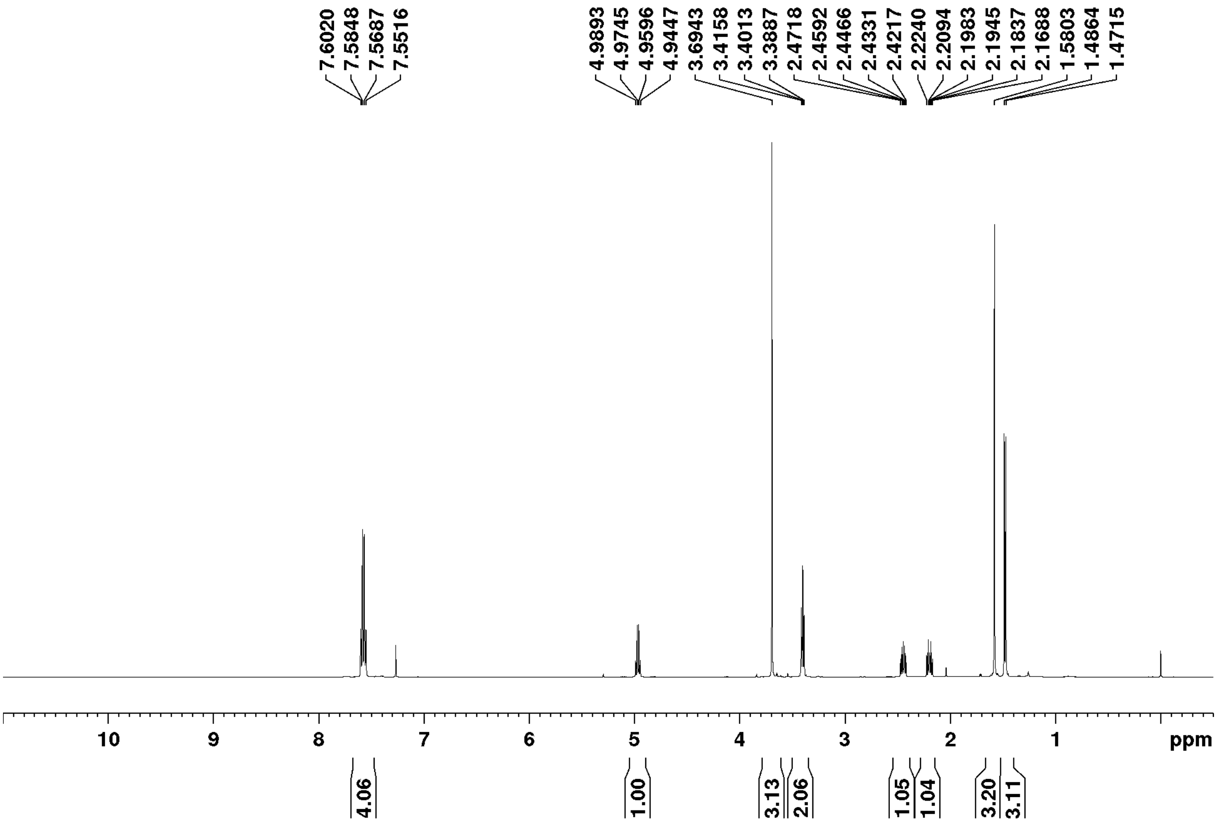

^{13}C NMR ($CDCl_3$, 125 MHz)

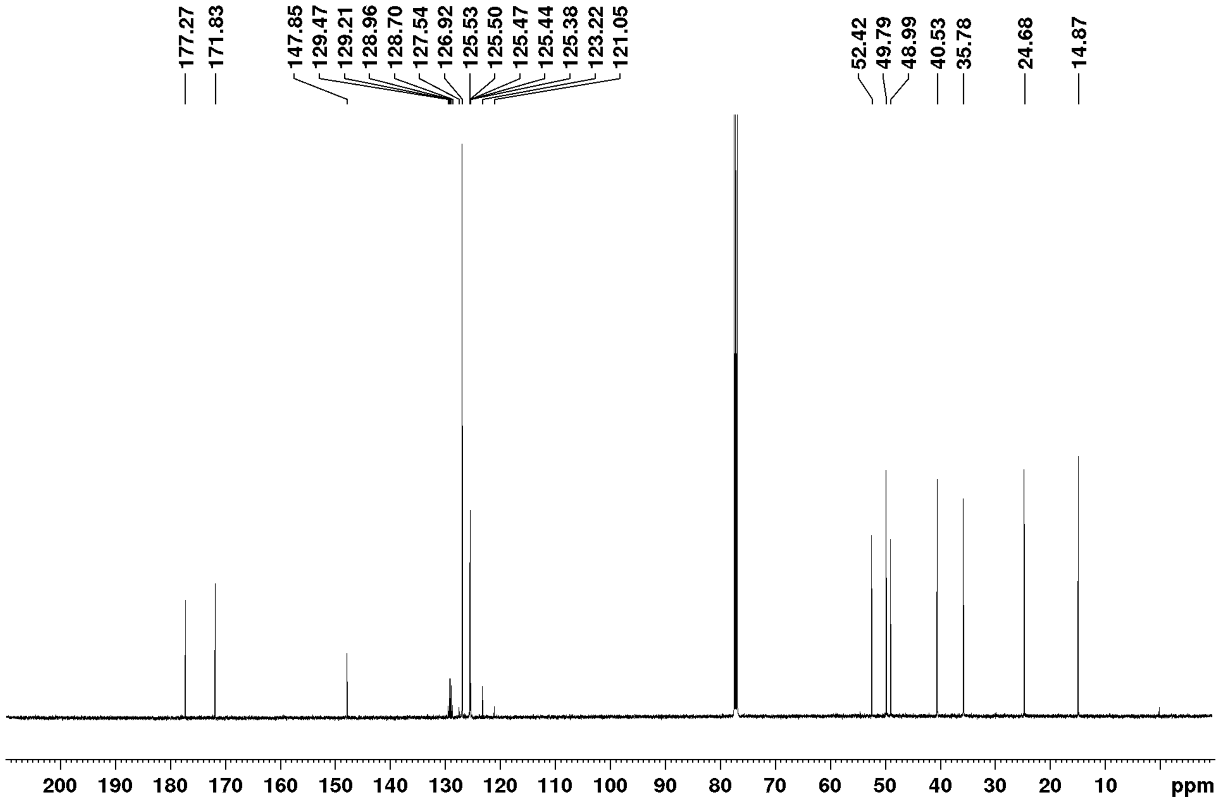

^{19}F NMR ($CDCl_3$, 470 MHz)

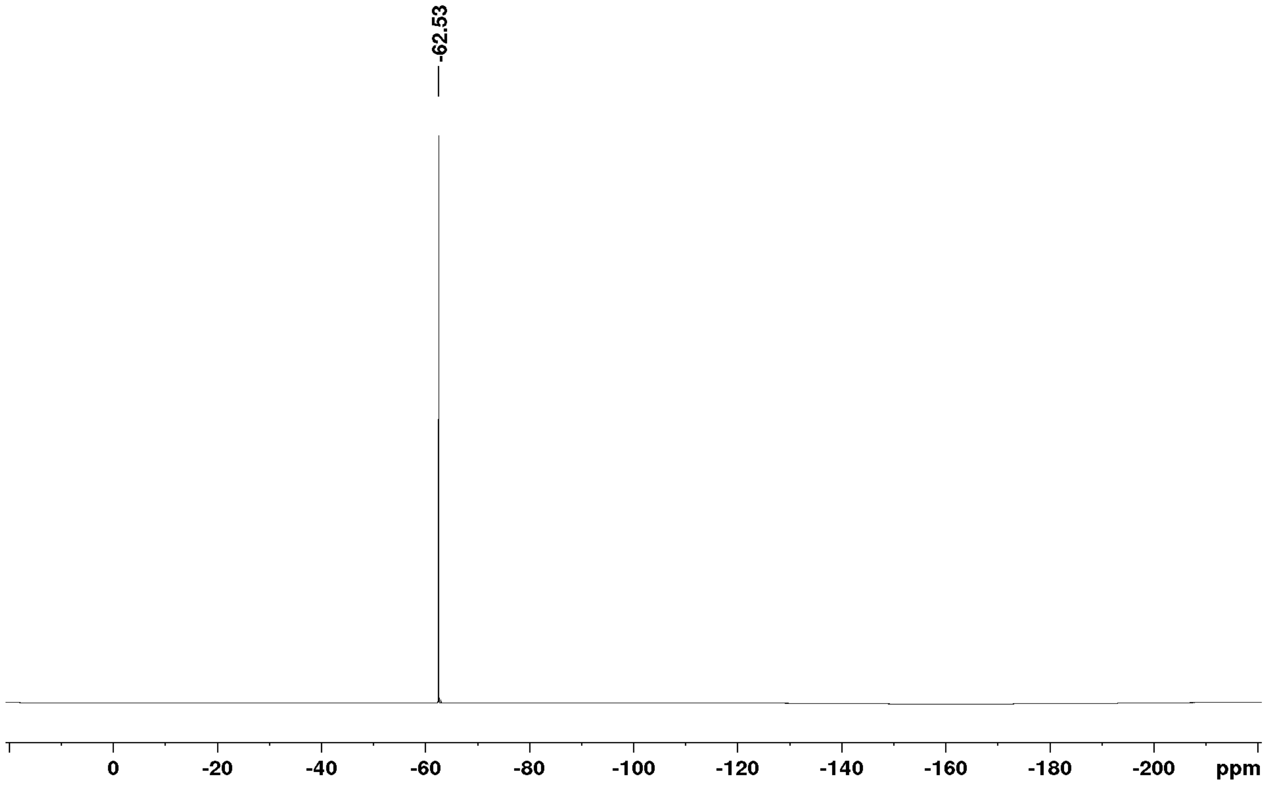

HRMS (ESI, positive) for $C_{16}H_{18}F_3NO_3$ (m/z): calculated 352.1131 $(M+Na)^+$, found 352.1128

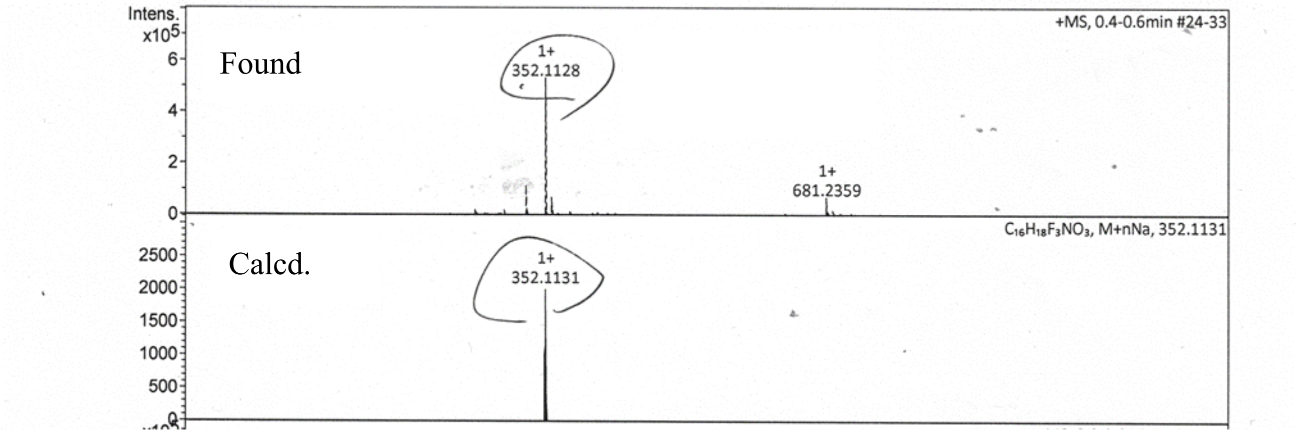

Methyl (*R*)-2-((*R*)-3-methyl-2-oxo-3-(4-(trifluoromethyl)phenyl)pyrrolidin-1-yl)propanoate (16')

^{1}H NMR ($CDCl_3$, 400 MHz)

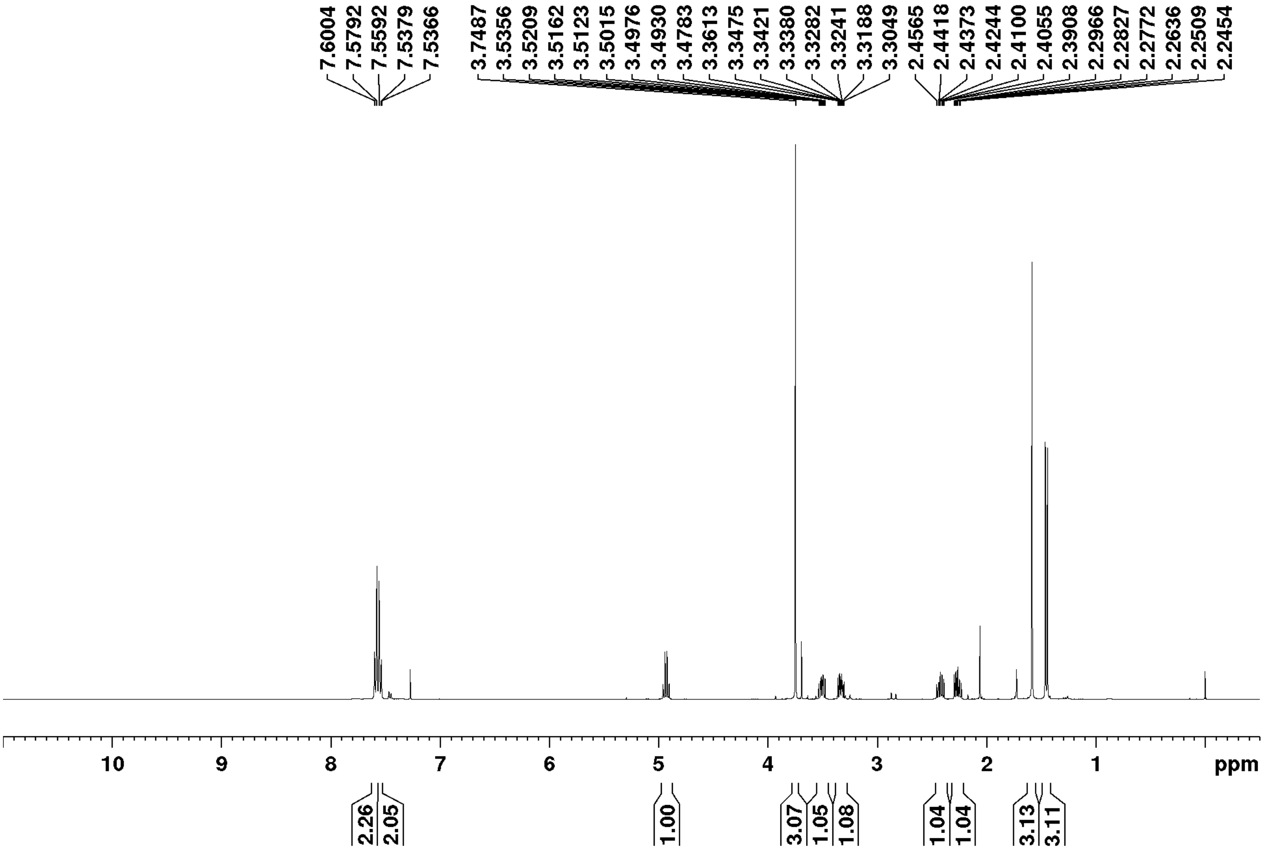

^{13}C NMR ($CDCl_3$, 125 MHz)

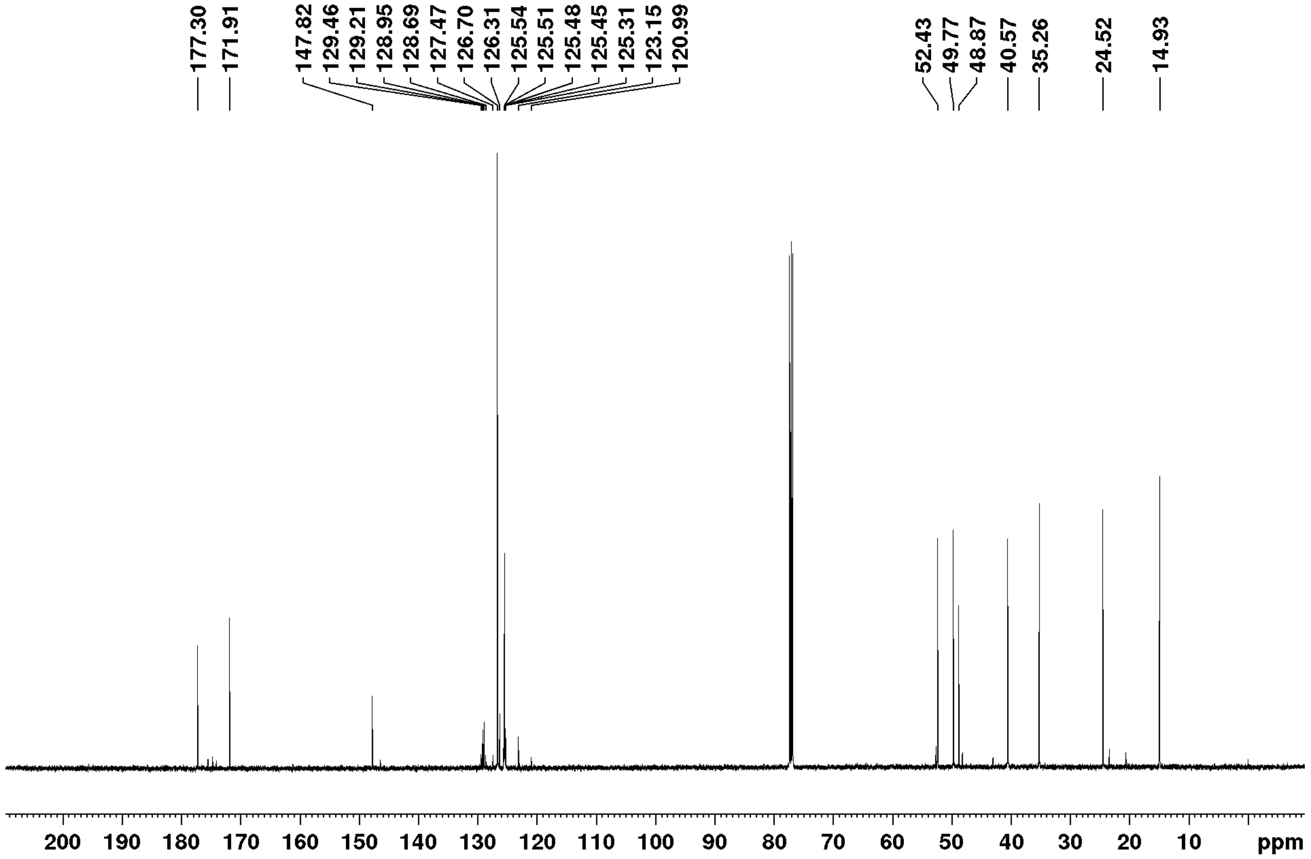

^{19}F NMR ($CDCl_3$, 470 MHz)

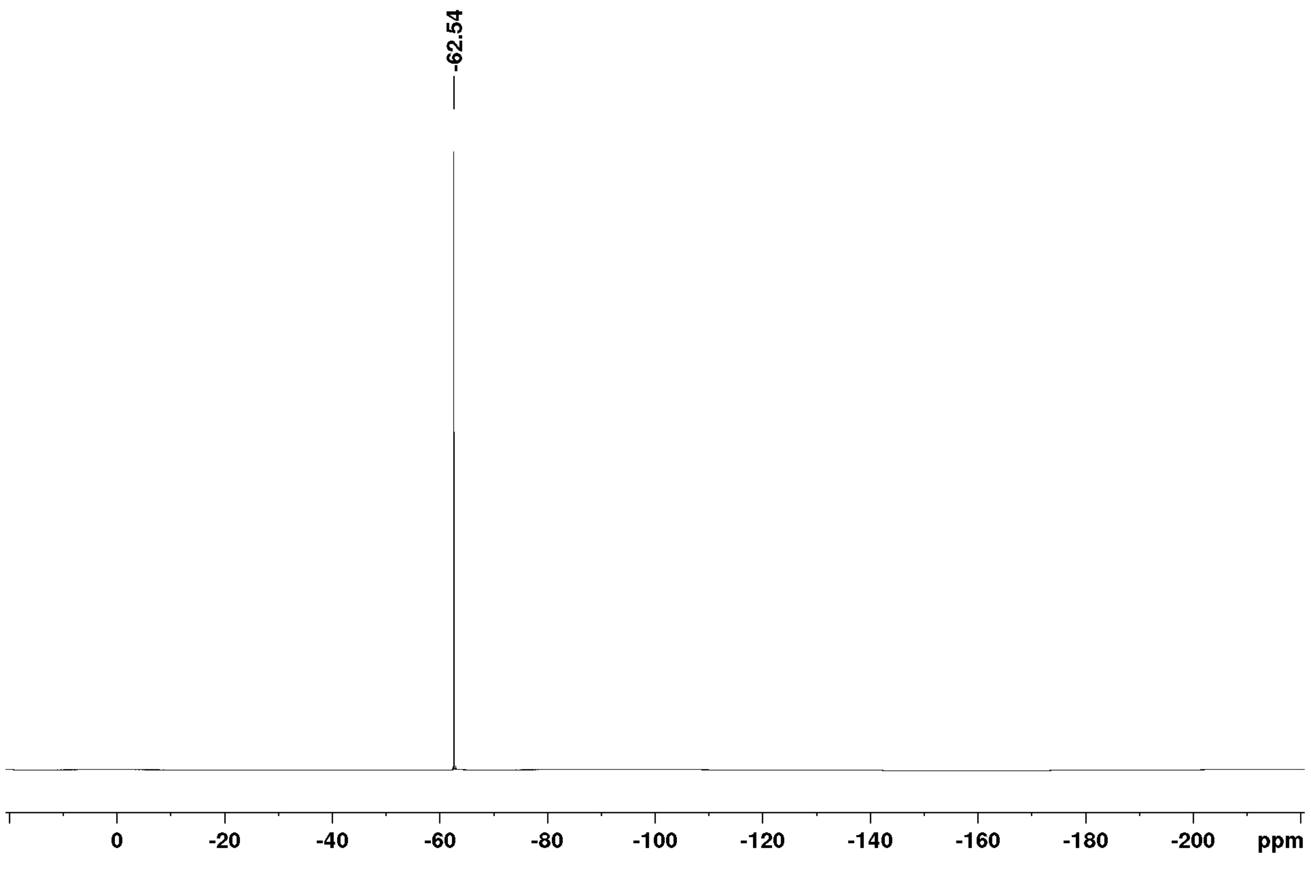

HRMS (ESI, positive) for $C_{16}H_{18}F_3NO_3$ (m/z): calculated 352.1131 $(M+Na)^+$, found 352.1145

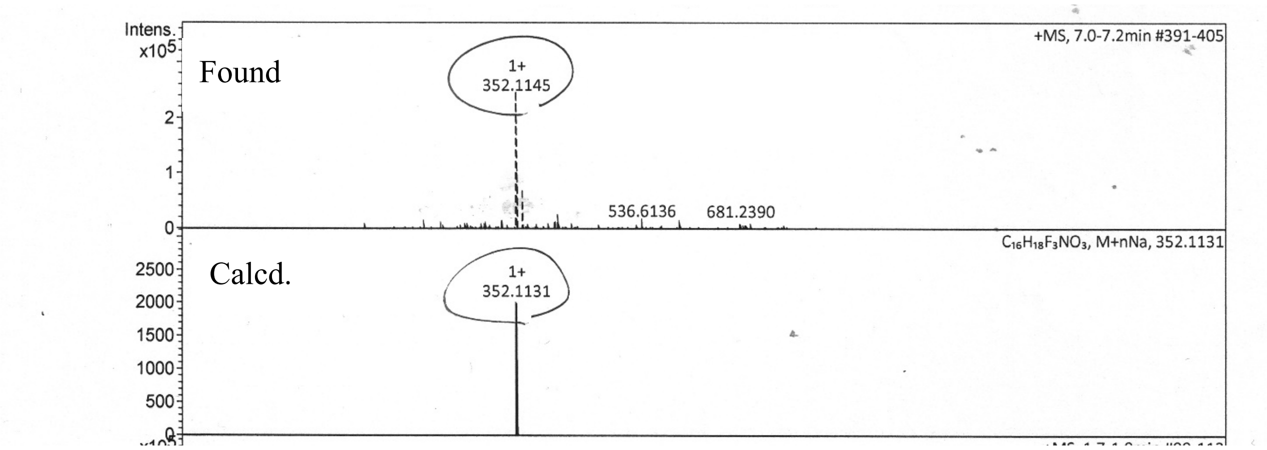

Methyl (*R*)-2-((*S*)-3-methyl-2-oxo-3-phenylpyrrolidin-1-yl)propanoate (17)

^{1}H NMR ($CDCl_3$, 400 MHz)

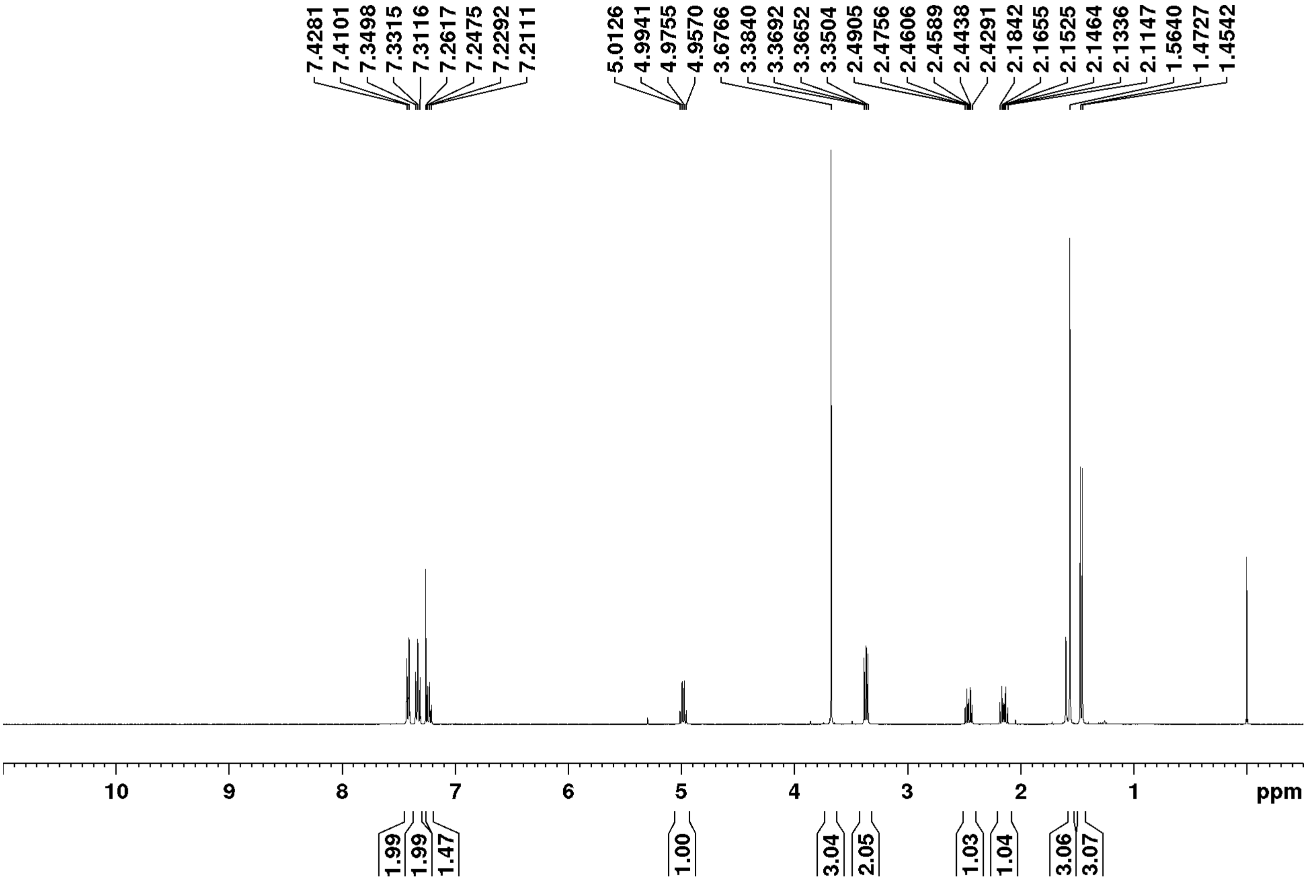

^{13}C NMR ($CDCl_3$, 125 MHz)

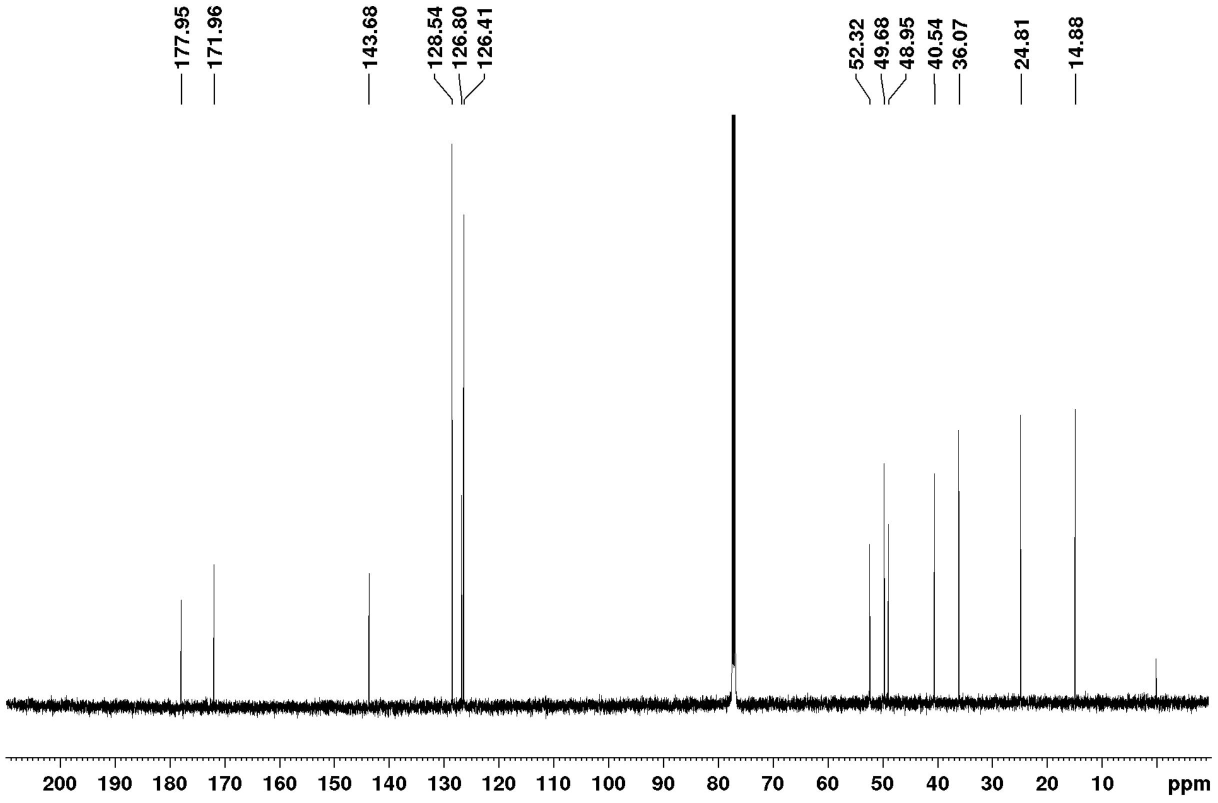

HRMS (ESI, positive) for $C_{15}H_{19}NO_3$ (m/z): calculated 284.1257 $(M+Na)^+$, found 284.1259

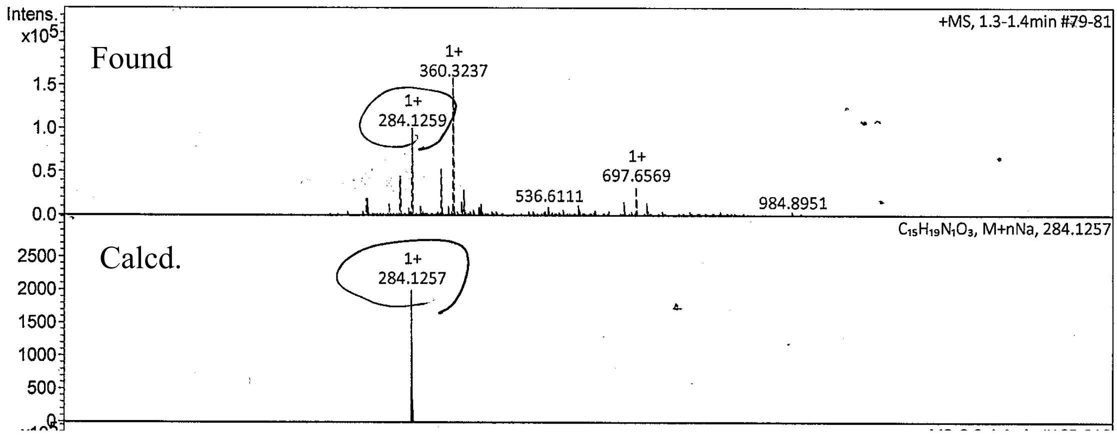

Methyl (*R*)-2-((*S*)-3-methyl-2-oxo-3-(3-(trifluoromethyl)phenyl)pyrrolidin-1-yl)propanoate (18)

^{1}H NMR ($CDCl_3$, 400 MHz)

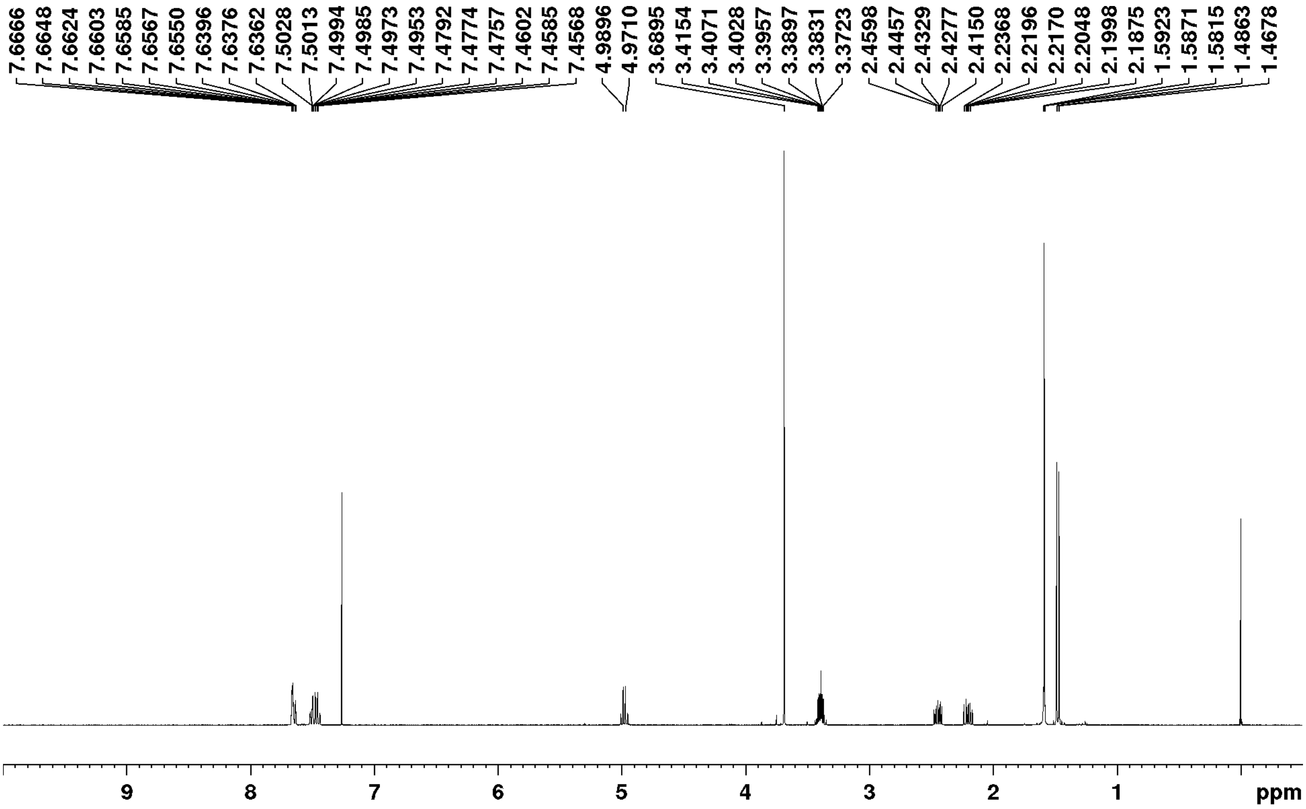

^{13}C NMR ($CDCl_3$, 125 MHz)

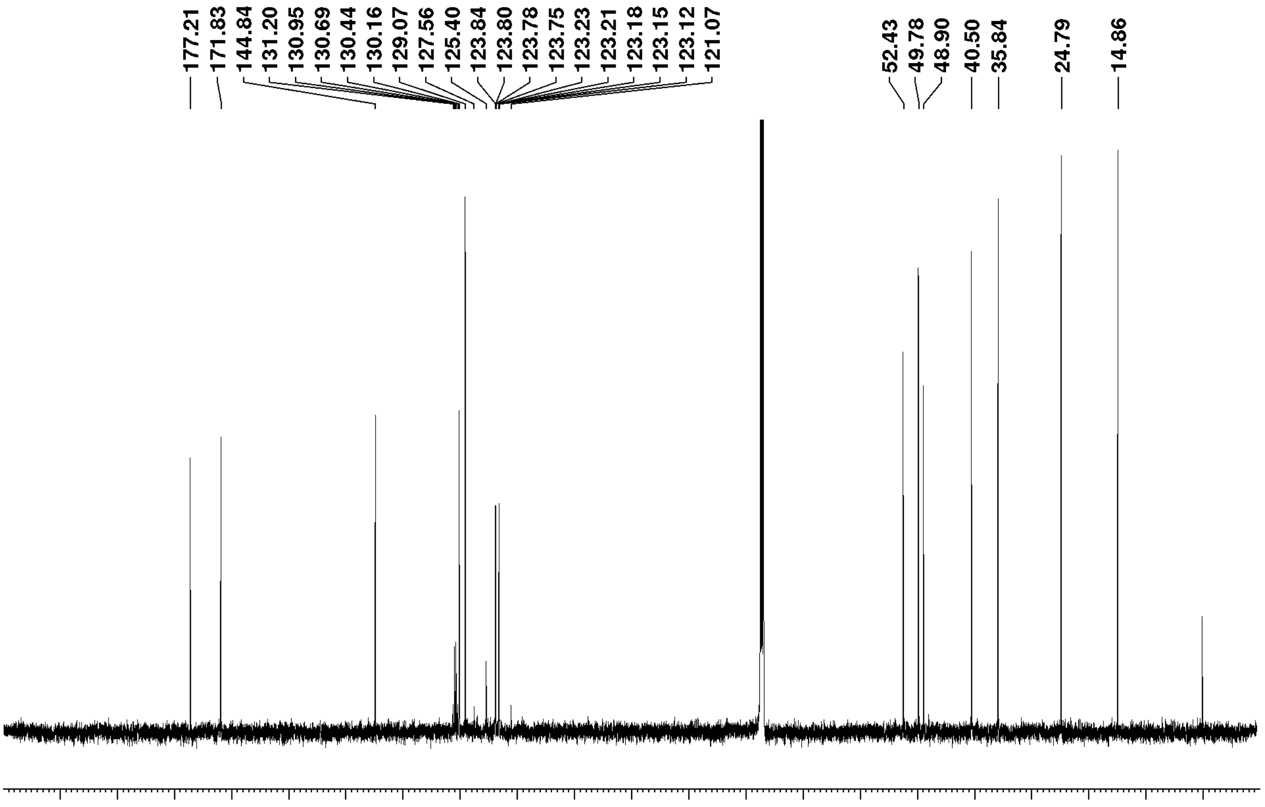

^{19}F NMR ($CDCl_3$, 470 MHz)

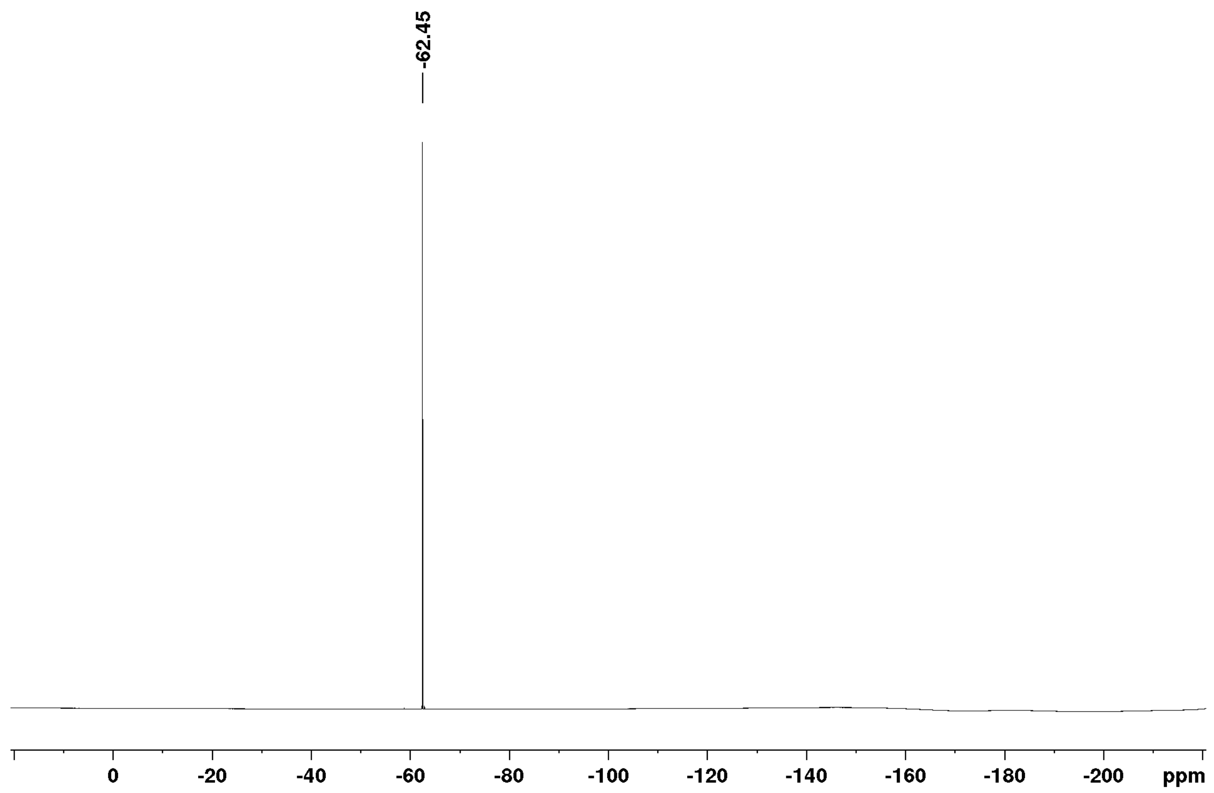

HRMS (ESI, positive) for $C_{15}H_{19}NO_3$ (m/z): calculated 352.1131 $(M+Na)^+$, found 352.1135

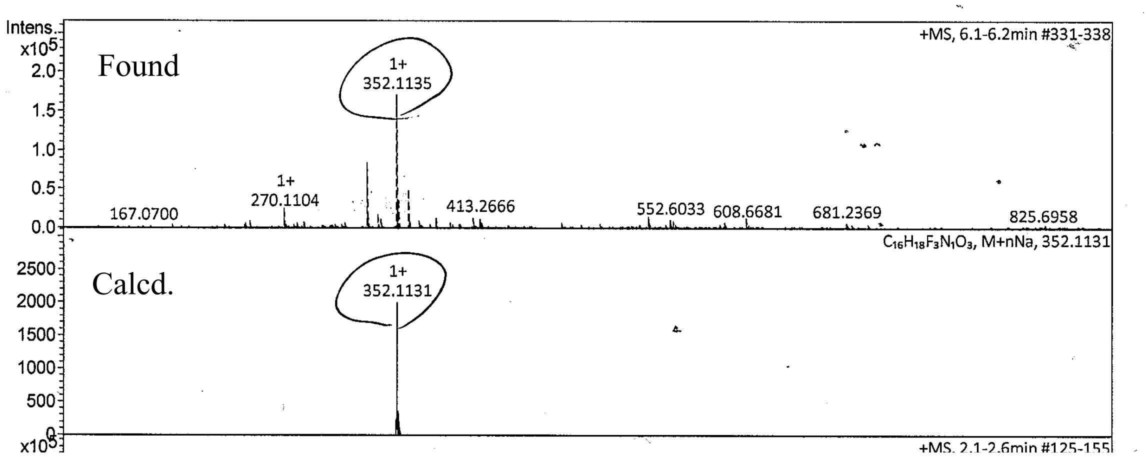

3. Purity analysis of tested compounds by HPLC

(*R*)-2-((*S*)-3-([1,1'-Biphenyl]-4-yl)-3-methyl-2-oxopyrrolidin-1-yl)-*N*-hydroxypropanamide (3)

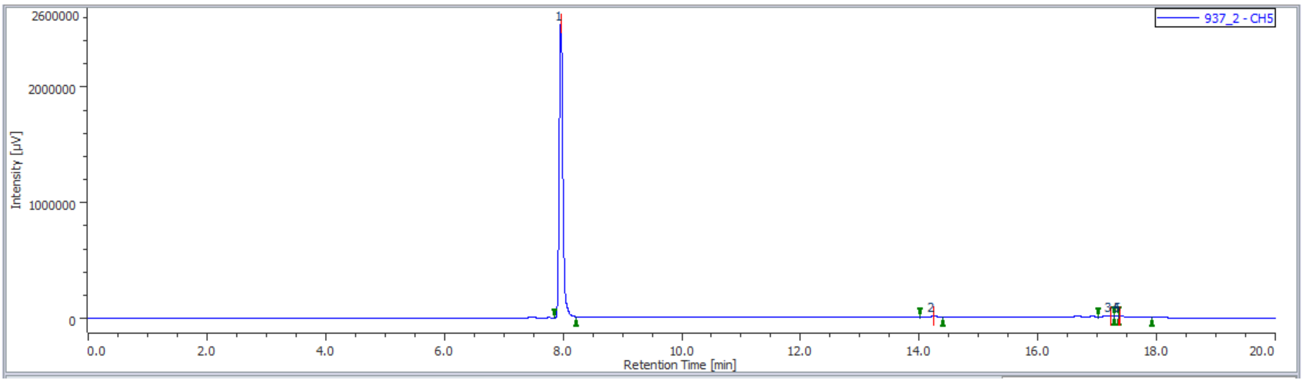

(*R*)-2-((*R*)-3-([1,1'-Biphenyl]-4-yl)-3-methyl-2-oxopyrrolidin-1-yl)-*N*-hydroxypropanamide (3')

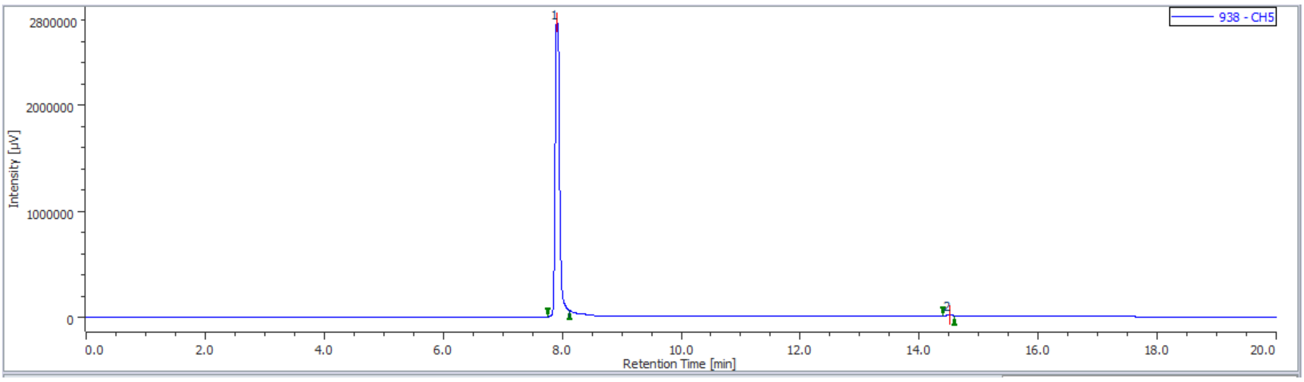

(*R*)-*N*-Hydroxy-2-((*S*)-3-methyl-2-oxo-3-(4-(trifluoromethyl)phenyl)pyrrolidin-1-yl)propanamide (4)

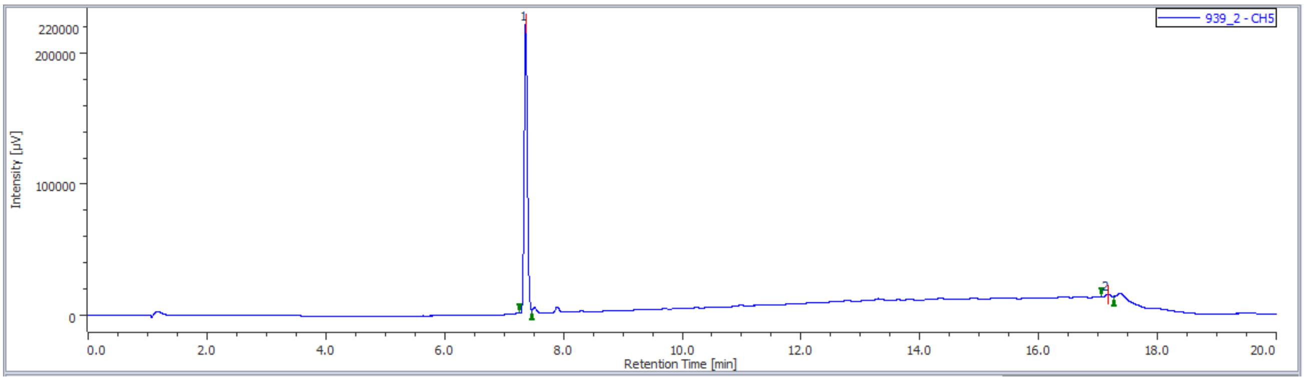

(*R*)-*N*-Hydroxy-2-((*R*)-3-methyl-2-oxo-3-(4-(trifluoromethyl)phenyl)pyrrolidin-1-yl)propanamide (4')

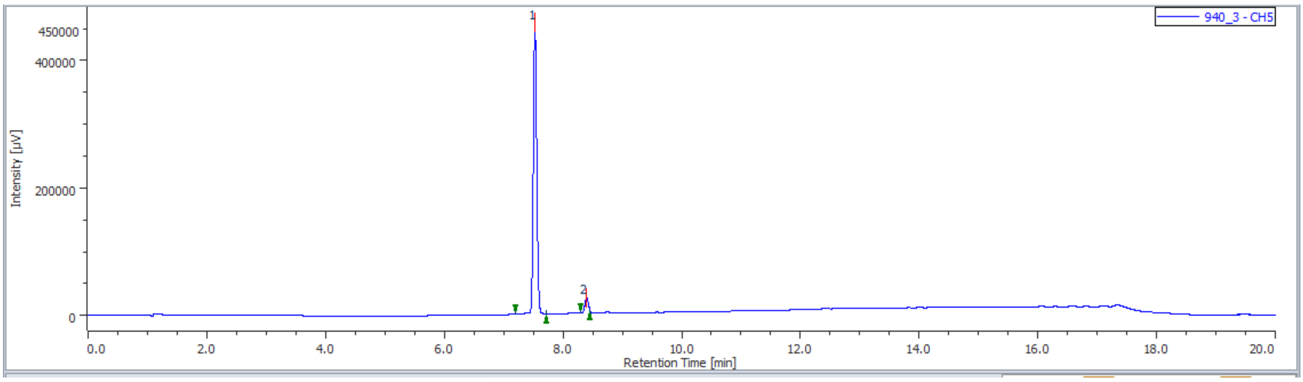

(*R*)-*N*-Hydroxy-2-((*S*)-3-methyl-2-oxo-3-phenylpyrrolidin-1-yl)propanamide (5)

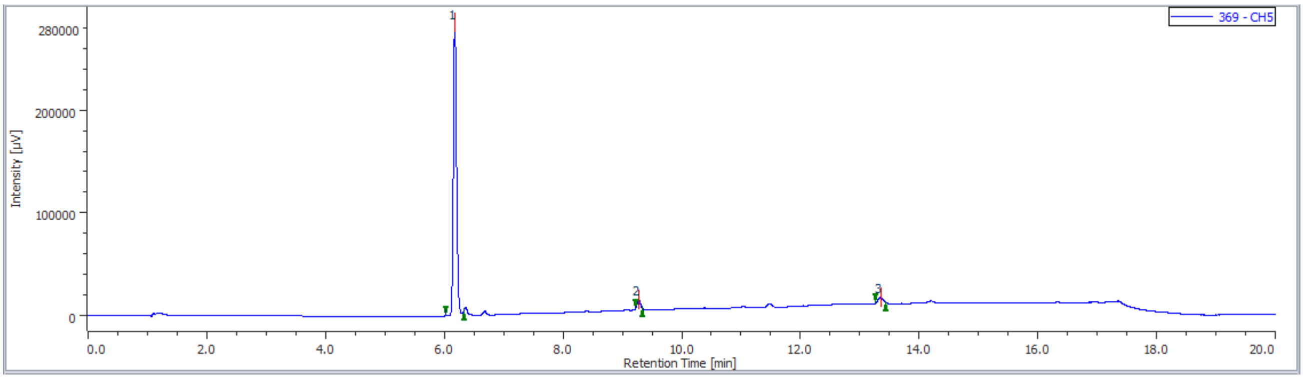

(*R*)-*N*-Hydroxy-2-((*S*)-3-methyl-2-oxo-3-(3-(trifluoromethyl)phenyl)pyrrolidin-1-yl)propanamide (6)

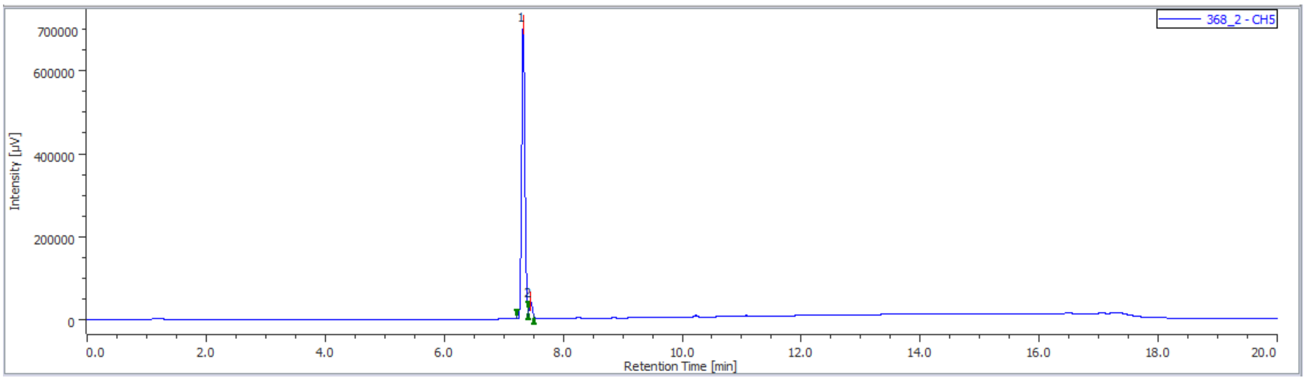

Supplement: Supplementary file 1 — Supplementary information. [file 41598_2020_71696_MOESM1_ESM.pdf]
